# Recent Advances in the Field of Bionanotechnology: An Insight into Optoelectric Bacteriorhodopsin, Quantum Dots, and Noble Metal Nanoclusters

**DOI:** 10.3390/s141019731

**Published:** 2014-10-22

**Authors:** Christopher Knoblauch, Mark Griep, Craig Friedrich

**Affiliations:** 1 Department of Mechanical Engineering-Engineering Mechanics, Multi-Scale Technologies Institute, Michigan Technological University, 1400 Townsend Drive, Houghton, MI 49931, USA; E-Mail: cjknobla@mtu.edu; 2 U.S. Army Research Laboratory, Weapons and Materials Research Directorate, Aberdeen Proving Grounds, MD 21005, USA; E-Mail: mark.h.griep.civ@mail.mil

**Keywords:** bacteriorhodopsin, quantum dots, noble metal nanoclusters, biosensor, molecular sensor, molecular electronics, bioelectronics, bionanotechnology, solar cell, fluorescent sensor

## Abstract

Molecular sensors and molecular electronics are a major component of a recent research area known as bionanotechnology, which merges biology with nanotechnology. This new class of biosensors and bioelectronics has been a subject of intense research over the past decade and has found application in a wide variety of fields. The unique characteristics of these biomolecular transduction systems has been utilized in applications ranging from solar cells and single-electron transistors (SETs) to fluorescent sensors capable of sensitive and selective detection of a wide variety of targets, both organic and inorganic. This review will discuss three major systems in the area of molecular sensors and electronics and their application in unique technological innovations. Firstly, the synthesis of optoelectric bacteriorhodopsin (bR) and its application in the field of molecular sensors and electronics will be discussed. Next, this article will discuss recent advances in the synthesis and application of semiconductor quantum dots (QDs). Finally, this article will conclude with a review of the new and exciting field of noble metal nanoclusters and their application in the creation of a new class of fluorescent sensors.

## 1. Introduction

Molecular sensing and molecular electronics is a diverse area that can include molecular conformational changes, changes in charge distribution, changes in optical absorbance and emission, or changes in electrical conductivity along or across simple or complex-shaped molecules, all in response to a target input. Each of these approaches can be integrated into a transduction system that provides a measurable and desired change in response to a specific or range of inputs. The ability to integrate such transduction mechanisms with biomolecules or to use biomolecules as the source of such materials provides, to varying extent, biocompatibility with other systems. In this review, we provide a summary of several biomolecular transduction systems and their applications. Specifically, the systems are bacteriorhodopsin and its integration with optoelectric sensing systems, semiconductor quantum dots and their application in sensors and electronics, and noble-metal nanoclusters.

## 2. Optoelectric Bacteriorhodopsin

Bacteriorhodopsin (bR) is an optoelectric protein found in the cytoplasmic membrane of the Archaean halophile *Halobacterium salinarum (H. salinarum)*. The protein is a light-driven proton pump that upon absorption of light at or near a wavelength of 570 nm translocates a proton from the cytoplasmic to the extracellular side of the cell membrane. This proton movement creates a charge difference across the membrane and has found use in sensor applications.

### 2.1. Bacteriorhodopsin Source

Bacteriorhodopsin is the bacterial analog of the visual rhodopsin found in the mammalian eye. bR is a 7-helix transmembrane protein that exists in a trimer configuration within the cell membrane, consists of 248 amino acids, and has a molecular weight of approximately 26 kDa [1]. The phospholipid cell membrane is approximately 6 nm thick and bR makes up approximately 75% of the membrane by weight. *H. salinarum* thrives and produces bR in high salinity and low oxygen environments. In its natural state, the proton pumping process is used as the energy source to drive the conversion of ADP to ATP for utilization by the cell. Through this proton translocation, a pH difference up to 4 across the membrane has been reported. This represents a charge ratio of 10,000:1 across a 6 nm thick membrane.

The host bacterium was thought to have been discovered in 1971 as *Halobactrium halobium* [2]. Prior to that, in 1940, the organism was apparently noticed and later named *Halobacterium Elazari-Volcani*. Through continued research it was found that several organisms with different names given by different researchers were indeed the same and collectively named *H. salinarum* in 1996 [3] with a strain designator of R1 [2]. A number of strain variants have been developed over the years to induce the cell to over-produce bR protein in the membrane.

When used for sensor applications, the cell is lysed and the contents digested and centrifuged to help in purification. The cell membrane is broken into fragments by sonication and these membrane fragments, each containing many bR molecules, are called Purple Membrane (PM) due to the royal purple color of the membrane patch. The membrane patches are typically 0.5 μm in the lateral dimension making them very flexible which allows attachment to irregular surfaces but also allows the patch to fold onto itself possibly reducing the net electrical effect of the proton charge translocation across the membrane. PM and bR are extremely robust and maintain function up to 80 C in solution, 140 C dry, pH from 2 to 12, are stable in most non-polar organic solvents, and in the dried state have a shelf life of years. These properties are the reason bR continues to be a material of interest for optical-electrical conversion applications such as sensors.

### 2.2. Bacteriorhodopsin Function

*H. salinarum* is a salt-loving extremophile bacteria and is cultured as such. The protocol described by Oesterhelt and Stoeckenius [4] has been widely used and modified for various bacteria strains [5]. Table 1 [5] shows the makeup of a typical growth medium. The medium should be well mixed to ensure dissolution of all reagents and the pH adjusted to 7.2 with 5 M NaOH. The medium must be autoclave sterilized and can then be stored at 4 °C. After inoculation with the bacteria, it is cultured in a sealed flask, under light with constant agitation, and at 40 °C. The logarithmic growth phase of the culture typically takes 5–7 days and can be followed using optical absorbance of the growth medium.

After culturing, the medium is centrifuged to isolate whole *H. salinarum* cells from the medium appearing as a red pellet. The pellet is then placed in distilled or deionized water which lyses the cells releasing the contents into solution. The coiled DNA causes the solution to become viscous. The DNA is digested using DNase 1 at room temperature using a stir plate for 24 h. Further centrifuging of this solution causes large cell debris to be separated and the supernatant is collected as it contains PM along with small cell debris. Centrifuging of the supernatant separates the PM from the lighter cell debris forming a purple to red color pellet while the yellow-color supernatant is discarded. This step is repeated until the supernatant is clear. Re-suspending this pellet in water produces a royal purple liquid which is introduced into a linear sucrose gradient for the final purification step. The purple band of the sucrose gradient is pipetted out and this material is the purified PM used for sensor applications.

### 2.3. Purple Membrane Deposition

For a pH in the range of approximately 5–12, the intracellular side of the PM patch is more electrically negative than the extracellular side. This provides an easy method for aligning and depositing the PM patches for sensor applications. The charge density, and therefore the electronegativity, can be changed by changing the pH of the suspension carrying the PM [6,7]. The presence and the ability to change the electrical characteristic are important for orienting and depositing PM by electrodeposition, Langmuir-Blodgett, or ionic self-assembly techniques.

#### 2.3.1. Electrodeposition

Because of the spatial charge difference on the two sides of the PM patches, they can be oriented by applying a DC-field to the suspension containing them. This not only orients the patches but also causes them to deposit on one of the electrodes with the more negatively charged cytoplasmic side toward the positive electrode [7]. While the method is easy and quick, there is no control over the number of layers deposited. The method is good for thick layers containing hundreds to thousands of PM layers [8].

The process uses two electrically conductive planar electrodes such as copper, brass, or indium tin oxide on glass which allows light to be transmitted to the deposited material. The parallel electrodes are typically spaced apart by approximately 1 mm using an elastomer O-ring placed on the bottom electrode. The suspension of PM in water is deposited within the O-ring forming a seal and the top electrodes placed on the O-ring and in contact with the suspension for electrical connectivity. Four V DC can be applied for 30 s to one minute which orients and deposits the PM onto the positive, bottom electrode. The excess water can then be pipetted away and the bottom electrode lightly rinsed. The PM adheres well enough while wet and the electrode can then be allowed to dry in air. It is best if drying takes place in approximately 50% relative humidity to keep the film from cracking and delaminating due to excessively rapid drying.

#### 2.3.2. Electrostatic Layering

Ionic attraction can be used to orient the dipolar PM [9,10] using poly(dimethyldiallylammonium chloride) (PDAC). The surface of the bottom electrode is first left with a negative charge by etching with dilute KOH. PM layers can then be built up by alternating incubation in either the PM suspension or PDAC solution with a wash and dry step after each incubation step. This process was used for up to 12 layers [10] but the orientation and number of monolayers was difficult to confirm.

#### 2.3.3. Langmuir-Blodgett Layering

The Langmuir-Blodgett (LB) technique provides a high degree of control over the orientation and deposition of PM onto a substrate. LB utilizes a sub-phase liquid on which a known amount of PM suspension is carefully applied with a syringe. Because of the lipid content and amphiphilic nature of the PM, the patches will orient themselves and spread into a thin disconnected layer. An LB trough has a fixed barrier and computer controlled moveable barrier to compress and maintain a uniform and unbroken PM layer. As a substrate is withdrawn up through the sub-phase and PM layer, the single layer of patches floating on the sub-phase adhere as a single layer on the substrate. A delicate measuring balance lifts the substrate while measuring the force. The output of the balance is used to drive the moveable barrier which compresses the PM layer on the sub-phase maintaining an unbroken film [11–20]. There are also variations to the basic process that combine LB with electrodeposition [21,22] and several horizontal deposition techniques [23–26]. While the LB technique provides excellent control over PM orientation and layer numbers, it is a necessarily slow process so that the PM film remains properly compressed by the barriers providing an unbroken film for deposition onto the substrate.

#### 2.3.4. Molecular Orientation Deposition

The bR protein spans the entire thickness of the cell membrane and extends slightly beyond both sides of the membrane. This exposes sites for binding other molecules to the bR, particularly one or more amino terminus on the extracellular side and carboxyl terminus on the intracellular side. These sites are well suited for binding biotin and streptavidin [27,28]. Biotin is a small molecule with a molecular weight of 244 Da (also known as vitamin B7) while streptavidin is a large molecule with a molecular weight of 53 kDa. Streptavidin has four binding sites for biotin and this conjugate forms one of the strongest covalent bonds found in nature with Ka∼10^15^ M^−1^.

The bR can be biotinylated at lysine 219 at a pH of approximately 9 [29,30] and forms the binding sites for streptavidin. Biotin can also be deposited onto a substrate by LB or other methods [31,32] and then incubated in a solution of streptavidin leaving exposed streptavidin binding sites. This construct is then incubated in PM patches with biotinylated bR which orients and strongly binds the patches. This technique also allows a finite density and number of PM patches due to the finite number of biotin-streptavidin binding sites [28,33–35]. In [32] this method produced a peak voltage output of 157.2 mV while non-oriented films produced 7.8 mV. Surface plasmon resonance was used to determine that 81% of the PM patches had the same orientation after binding.

### 2.4. Purple Membrane Patterning

Several methods for patterning purple membrane on a substrate have been reported. These methods generally fall into one of two processes: deposition of a broad film of purple membrane followed by an energetic removal process; or by a lithographic process whereby purple membrane is selectively deposited or removed by a chemical process.

#### 2.4.1. Patterning by Energetic Removal

Laser ablation has been shown to pattern a broad film of purple membrane. Haronian and Lewis [36] first deposited a film of PM onto a conductive gold coated substrate. Then using an electron microscope grid they exposed the film to an ArF excimer laser [37] which ablated the PM from between the parallel 30 μm openings of the grid. After the grid was removed gold was sputtered creating a conductive top layer. The parallel grid was reapplied perpendicular to the original direction and again laser-ablated creating an addressable array of 30 μm square pixels.

**Figure 1.**
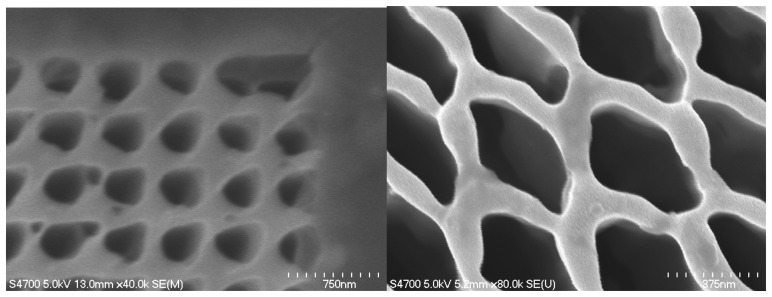
Electrodeposited PM patterned by FIB [38].

Anton [38] used a 30 keV focused ion beam (FIB) to create a pattern in a film of PM first deposited by electrodeposition. The FIB system used a CAD-type beam raster generator with programmed current and pixel dwell time to selectively remove the PM as shown below (Figure 1). The left figure shows a regular square array of holes where PM was removed. The right figure shows a more irregular but deeper and more well-defined pattern. One drawback to this method is that gallium will be deposited within the PM that can change or destroy its photoelectric function. However, Monte Carlo modeling estimated that the penetration depth of the ion beam in the PM extended to approximately 60 nm with the bulk of the deposition occurring at approximately 40 nm depth with 20 nm of lateral scattering.

#### 2.4.2. Non-Energetic and Lithographic Patterning

Several processes for patterning PM without exposing it to high energy have been demonstrated. Libertino [39] patterned PM on silicon by first thermally growing a 25 nm thick silicon dioxide film on a silicon wafer, followed by a 200 nm thick layer of polysilicon. This was then masked and plasma etched down to the silicon dioxide. The mask had openings and features ranging from 600 nm to 2 mm. The PM was allowed to deposit onto the oxide in the bottom of the openings in the polysilicon layer by soaking in a suspension of PM and deionized water for 10 min and then drying with nitrogen. By this method, the PM patches in the layer would not have been directionally oriented. A photoresponse measurement was not reported.

Crittenden [40] demonstrated a soft lithography process using a gold coated polydimethylsiloxane (PDMS) master resembling a microfluidic channel geometry. After the PDMS master was patterned, it was coated with 70 nm of gold. Adhesive tape was then applied to the master and removed taking the gold from the top portion of the lands between the channels. This left gold on the bottom and sidewalls of the PDMS microchannel master. The PDMS master and an ITO-coated substrate were then pressed together and a suspension of PM in water was injected into the channels of the PDMS master. Approximately 5 V DC was applied for 5–30 s between the gold and ITO surfaces providing for electro-orientation and deposition of the PM. The patterned PM was rinsed and dried. Through scanning force microscopy it was shown that the PM was deposited and patterned but a photoresponse was not measured. It was estimated that there were 10 monolayers of PM deposited.

Anton [5] demonstrated a lift-off optical lithography process for PM that was applied to conductive and non-conductive substrates, and was used to modulate MOSFETs with light as will be described in the next section. In this process, if the substrate was non-conducting then a gold or chrome-gold layer was applied to create a conductive layer. Then similar to standard MEMS-type processes, photoresist was spun onto the substrate and patterned by optical lithography using a chrome mask. After development of the resist, openings remained that extended down to the conductive layer. A thick film of PM suspension in deionized water was deposited over the area of interest and an ITO-glass wafer placed as the top electrode. Standard electrodeposition was performed immobilizing the PM within the openings in the patterned resist, followed by drying. The photoresist and remaining PM film was removed by a brief acetone soak that dissolved the resist and with it removed any PM on the resist. It is important to note here that PM is quite stable in acetone so the PM that was electrodeposited in the resist openings remained. The reference also provides results of typical lateral features down to 5 μm.

### 2.5. Purple Membrane Applications

The durability, patterning options, and photoelectric properties of bR in PM make it a very useful transduction material for electronic and molecular sensing applications. The three applications described are for bio-based solar cells, transistor modulation by light, and as a sensor medium where the bR action is modulated in the presence of a molecular target. However, the traditional process for extracting and purifying PM is slow and therefore relatively expensive requiring equipment and knowledgeable technical support. Shiu *et al.* [41] describe a process to reduce a substantial portion of the time and processing requirements to purify PM. The first several process steps remain the same up to and including lysing of the cells and digestion with DNAse. In the traditional process there would be several steps of ultracentrifuging and then final separation across a sucrose gradient. Shiu *et al.* demonstrated using aqueous two-phase separation (ATPS) to eliminate the relatively delicate sucrose gradient step. In ATPS, the cell lysate after the DNAse step is mixed with potassium phosphate solution, dibasic and a PEG 8000 solution for one hour. The aqueous phases were then separated by a 15 min centrifuge forming the purple band that was extracted similar to when using a sucrose gradient. The extracted PM in DI water was centrifuged for 30 min and designated ATPS1. An additional 15 min centrifuge step removed additional debris and was noted as ATPS2. This process reduced the PM extraction process from approximately 27 h after cell lysis to approximately 2.5 h. In their optical absorption measurements, ATPS2 was approximately 2.5% less pure and ATPS1 8% less pure than that from a sucrose gradient. The bR in PM yield measured by hydroxylamine bleaching was slightly higher in both ATPS approaches compared to a sucrose gradient. This newer process could be used to help reduce the cost of PM for larger applications.

#### 2.5.1. Bio-Based Solar Cells

Because bR is a proton pumping protein dispersed in the purple membrane it has been shown to be operable in a bio-based solar cell. There have been a number of reported studies however for brevity three will be mentioned here. Bertoncello *et al.* [22] looked at the potential of using PM and compared it with other solar scavenging technologies including silicon, gallium arsenide, indium phosphate, and several polymer based technologies. Their analysis showed that light sensitive proteins might operate with a maximum theoretical conversion efficiency of 25%, the highest of the technologies that were compared. This was calculated by comparing the work of charge translocation through the cell membrane with the incident photon energy. Their analysis also suggested an average specific power of 250 W per square meter and average specific power density of 2103 W per kilogram. These values were predictions and not demonstrated by operating solar cell-type structures.

Thavasi *et al.* [42] demonstrated a bio-sensitized solar cell (BSSC) similar in structure to the more widely explored dye-sensitized solar cells (DSSCs). They used FTO-coated glass as the basic electrode structures. The working electrode was coated with nanoparticles of TiO_2_ upon which PM was immobilized. The counter electrode had a thin aluminum film. The electrolyte was lithium-iodide and iodine in a KCl buffer at pH 8 similar to that used in DSSCs. The output of the cell produced a short-circuit current of 0.089 mA per square cm and an open-circuit voltage of 0.35 V under broad spectrum excitation of 40 mW per square cm.

Al-Aribe *et al.* [43] described dry and wet PM films used in a photovoltaic cell. They used a self-assembled monolayer with biotin binding on a gold coated substrate. The PM monolayer was covered with an ITO electrode for the dry cell. The wet cell used a gold-coated porous substrate between two reservoirs with KCl. The dry cell showed a response of 9.73 mV per square cm and the wet cell showed 41.7 mV and 33.3 μA, both per square cm.

Others have shown that the photoelectric output of bR can be enhanced by scavenging more of the solar spectrum than only at the absorption peak of bR at 570 nm. Yen *et al.* [44] investigated such an increase by incorporating a Nafion membrane to pass the expelled proton from the bR, and by selecting and capping a specific nanoparticle to enhance the blue absorption of the longer-lived M-intermediate of the normal bR photocycle. This part of the bR photocycle is responsible for the reprotonation back to the ground state and this enhancement can result in a larger current. In this work, they found that a 40 nm cuboid silver nanoparticle capped with 40 nm thick poly(vinylpyrrolidone) (PVP) with a molecular weight of 55,000 gave the highest stable current and largest spectral overlap with the M-intermediate absorption. The current density was 0.2 uA per cubic cm and was stable for up to 6 h of continuous illumination giving a reported 50-times higher current density than pure bR in a wet electrochemical system.

Karna *et al.* [45] used semiconductor quantum dots (QDs) bound to the PM by the biotin-streptavidin system. QDs were chosen to scavenge blue light and radiate it at approximately 570 nm for use by the bR photocycle. Open circuit voltage measurements showed a 35% increase compared to PM without attached QDs. The voltage with QDs was approximately 0.3 V in a system with a glass substrate, coated with ITO and either TiO_2_ nanotubes or ZnO nanowires, then PM. 0.1 M KCl was the conductive medium. This work showed a second method of scavenging short wavelengths using bound quantum dots and a system that has been adapted for sensing applications as will be shown subsequently.

#### 2.5.2. PM Integration with Electronics

There have been demonstrations of using PM to modulate the throughput of conventional transistors and nanotransistors by having the PM act as the gate input to the devices. This shows a further integration of the material for optoelectronic and/or chemo-electronic sensors. Somewhat similar applications have been demonstrated using PM in optical detectors. Fukuzawa [46] for example, took advantage of the fact that the M-intermediate of the bR photocycle can be greatly delayed using the PM in a high-pH environment. Normally with broad spectrum illumination the 570 nm light initiates the photocycle with 410 nm light returning the bR to the ground state from the M-intermediate state. In this work, a rectangular PM area, 4 mm × 15 mm was made by eletrodeposition. The PM had been treated with a borate buffer of pH 9 prior to drying to delay the transition from the M-state back to the ground state. Two electrodes were imparted to the rectangle by using two triangular top electrodes that were electrically isolated from each other. A narrow vertical 4mm stripe of pulsed 570 nm light was moved across the two electrodes. The variable photovoltage in each triangular top electrode was measured and an algorithm was used to sense the location of the stripe as it moved with good agreement between actual and measured positions. This demonstration showed an example that PM can be used in dynamic optical applications such as one- and two-dimensional photodetectors.

There has been a body of work showing that PM can be integrated with transistors to modulate those devices under variable illumination. Xu *et al.* [47] and Bhattacharya *et al.* [48] describe the integration of PM onto the gate electrode of GaAs field-effect transistors. With 594 nm light from a He-Ne laser variable from zero to approximately 70 mW per square mm, the device showed a response of 3.8 A/W of excitation. Later, Xu *et al.* [49] demonstrated a similar application using an InP-substrate and device architecture of InGaAs/InAlAs with oriented PM on the gate. In this case the extended Ti/Au gate was 1mm diameter and the PM was 50 um thick. Under variable optical power of 594 nm wavelength, the device showed a response of 175 V/W of optical power and a 16 dB dynamic range. Shin *et al.* [50] showed an n-channel silicon MOSFET with PM on the extended Ti/Au gate electrode with a biasing capability. Using variable power 594 nm light a response of 4.7 mA/W was shown.

Anton *et al.* [51,52] demonstrated the photolithographic lift-off process to integrate PM onto extended Au gates of conventional MOSFETs. In this work the extended gate pads varied from 250 μm square to 4 mm square. This work showed the extreme sensitivity possible with this type of architecture. A yellow LED array was used for illumination with optical power measured specifically at 570 nm. At a LED-device distance of 300 mm and an optical power of 1.6 μW per square cm, the induced photovoltage of the PM was measured to be 0.5 mV and the MOSFET current throughput was 0.5 nA. Even at these extremely low values, the current waveform correlated with the flashing of the LED array.

Pushing the device sizes even smaller, Walczak [32] and Walczak *et al.* [53,54] integrated PM onto the gates of multiple quantum dot-based room-temperature single electron transistors (SETs). In this work, the SET devices were fabricated using focused ion beam deposition of 8 nm and larger tungsten quantum dots in the active area between larger electrodes. The PM was illuminated with a yellow LED array with a flash period ranging from 2.5 s to a very long 83 s. In each case the change in SET drain current was approximately 0.6 nA for 2.5 s to 1 nA for 83 s. An electrical analogy model was also presented for these devices from measurements taken of resistance and capacitance of the PM under changes of illumination intensity. There was good agreement between the model and measured PM photovoltage.

#### 2.5.3. PM for Sensing Applications

The utility of PM-based sensors has also been shown. For example, Sharkany *et al.* [55] showed several methods for using the changes in optical properties of PM in the presence of relative humidity and the presence of ammonia. In the former work, they dispersed PM into a gelatin with triethanolamine added for greater photosensitivity and then deposited this onto glass slides or the end faces of 600 μm quartz fibers. The changes in PM absorption were measured at 412 nm and 570 nm with a spectrometer with 570 nm LED illumination. For changes in relative humidity (RH), the reported 412 nm absorption changes varied from approximately 0.17 at 12% RH up to approximately 0.37 at 90% RH after which the change decreased. In the presence of ammonia, the optical transmission of 412 nm light increased by approximately 35% compared to an absence of ammonia.

Korposh *et al.* [56] later showed the change in absorbance changes in ammonia concentration from zero (air only) to 10,000 ppm using optical fibers with an exposed end covered with a PM, gelatin, and triethanolamine mixture as above. They showed static conditions where the absorbance change stabilized after 120 s and also dynamic changes where the test chamber was exposed to increasing ammonia concentrations with 50% RH air introduced periodically to flush ammonia from the chamber. In all cases the response generally showed a first-order response with a reported detection limit of 5 ppm.

Other applications of PM for sensing have expanded to methods for stopping or starting the bR photocycle and subsequent photovoltage in the presence of a target species. Griep [57] and Griep *et al.* [58–62] developed a method whereby streptavidin-coated semiconductor quantum dots are attached to a biotinylated site of the bR just above the membrane lipid bilayer. The quantum dot is selected so it will fluoresce near 570 nm when illuminated with UV light which did not initiate the bR photocycle. A dark quencher molecule was attached to the QD with a very short linker molecule allowing energy to be non-radiatively transferred from the QD to the dark quencher via FRET coupling. The dark quencher was chosen such that the target molecule of interest preferentially displaced the dark quencher if the target was present in the environment. Under conditions with a lack of target, the bR activity does not operate due to a lack of proper illumination as the 570 nm output of the QD is quenched. In the presence of the target, the linker is severed permitting the QD to fluoresce and initiating the photocycle with a measurable output. This was shown to be effective for a system with maltose binding protein where the quencher was displaced in the presence of maltose. A 1mM concentration of maltose was demonstrated.

Winder [31] and Winder *et al.* [60,63] showed a similar architecture and the selectivity to the target. In this work, a maltose binding protein and PM hybrid was constructed. The addition of sucrose to the environment did not elicit a response, but a 175 mV change in the hybrid sensor was observed with the addition of 1 mM of maltose. This work shows the utility of both a PM/bR hybrid with a target-specific linked molecule and that the hybrid can have excellent selectivity to the target.

## 3. Semiconductor Quantum Dots

The development and application of QDs has accelerated rapidly in recent years, providing highly tunable nanoscale materials with applicability towards a wide-range of engineering fields. Although the toxic materials used to synthesize semiconductor QDs make them unsuitable for biocompatible applications, their unique properties make them ideal for a multitude of sensing applications. One highly sought after characteristic for semiconductor QDs in particular are their optical properties. Semicondunctor QDs have the innate ability to absorb photons with energy over a wide range of the spectrum from ultraviolet to the visible and exhibit bright, atom-like narrow emission bands in the visible that can be further tuned by changing the size or composition of the particles [64–66]. With the electron and hole pair confined in all three dimensions the bandgap can be controlled through manipulation of the QDs diameter, with a smaller crystal size producing a higher energy bandgap. The tunability of the semiconductor QDs bandgap relies both on the material composition and the diameter of the QD colloid, typically ranging from 2 to 10 nm. Assembled as both core and core/shell structures of mixed metal dichalcogenide and alloyed structures, they can be easily tuned to fit energy spectra spanning from the UV to nIR wavelengths and extremely high quantum yield efficiencies.

The innate properties of QDs make them desirable source of photons for the study of the photo-assisted structural changes and dynamics of biomolecules [67–69]. Furthermore, these QDs exhibit exceptionally high chemical and physical stability, low-photobleaching, and the ability to bind with both organic and biomolecules. These characteristics in conjunction with their nanoscale dimensions and ease of surface functionalization have made them a prime material for applications in the biomedical field including drug delivery, biosensors, cellular targeting, biolabeling, therapeutics, and fundamental biological studies. Even with their impressive properties, however, a gap in the fundamental understanding of how these engineered QDs interact with biomolecules and in a biological environment has proven to be a major obstacle to the implementation of QDs in biomedical applications. The interactions are complex, involving a wide-array of variables including the basic properties of the QD (size, capping agents, surface structure, shape, atomic composition, crystallinity, *etc.*), type of biomolecule (enzymes, DNA, proteins, organelles, *etc.*), effects of suspension environment (pH, ionic strength, polar/nonpolar, temperature, stability, charge states, *etc.*), and the dynamic behavior of the formed interfaces. With the trend towards *in vivo* QD sensor applications, additional emphasis towards understanding the role of ligand length, charge, and QD size on cytotoxicity must be considered [70,71].

### 3.1. Quantum Dot Bioconjugation

**Figure 2.**
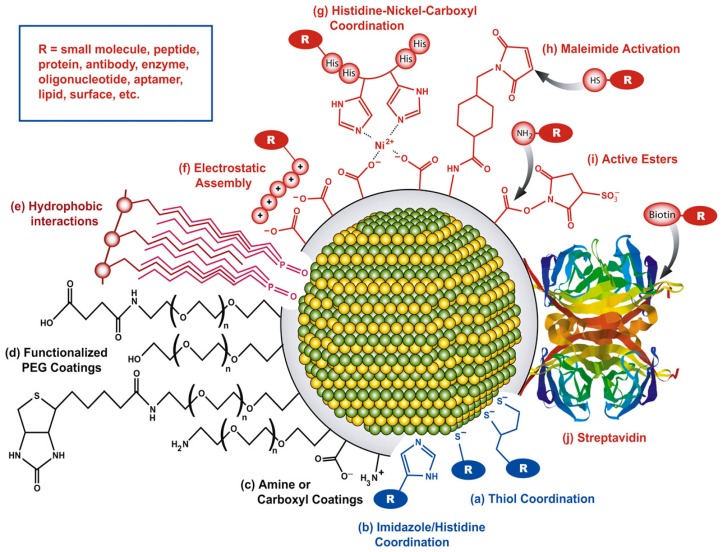
Bioconjugation schemes for QD functionalization including (**a**) thiol coordination; (**b**) polyhistidine groups; (**c**) amine/carboxyl moieties; (**d**) PEG; (**e**) hydrophobic interactions; (**f**) electrostatic adhesion; (**g**) nickel mediated polyhistidine; (**h**) maleimide covalent linkage; (**i**) active esters; and (**j**) biotin-streptavidin complexes. Figure used with permissions of publisher [73].

To take advantage of the unique properties of QDs in a diverse array of sensor applications and environments, careful selection and preparation of the functional surface coatings must be addressed. Depending on the application environment, biomolecule of interest, and desired sensing mechanism, numerous QD bioconjugation strategies exist as shown in Figure 2, including direct covalent linkages, polyhistidine-metal-affinity coordination, electrostatic adhesion, and chemoselective ligation [72].

The proper application of these varying conjugation schemes provides a pathway to control critical criteria of the biomolecules attachment including valency, affinity, biomolecular orientation, QD-biomolecuar spacing, and orientation of the target binding spot. Biomolecular orientation with proteins, for example, can take advantage of the terminal amine and carboxyl groups for site directed attachment on the QD via carbidomide linkage chemistry. Similar methodologies can be utilized with synthetic oligonucleotides and aptamters, and can utilize broader linkage chemistries at a site-specific locations on the biomolecule using thiol, polyhistidine, and biotinylation (for biotin-streptavidin conjugation) [73].

Another common conjugation strategy that allows control of spatial separation and material orientation is electrostatic layer-by-layer (LBL) deposition. As mentioned previously, by controlling the surface charge on the materials, to ideally be greater than ± 30 meV, monolayers can rapidly be adsorbed to oppositely charged surfaces. The LBL layering strategy has proven to be a common methodology employed to achieve QD biofunctionalization in sensor applications [62,74,75], with monolayer formations comparable to that of Langmuir-Blodgett deposition [76]. The ease of deposition of QD systems has made sensor platforms feasible in both the aqueous suspension and solid substrate forms, allowing for the utilization of the inherent QD sensor mechanisms in virtually any environment.

### 3.2. Quantum Dot Sensor Mechanisms

The optimization of QD synthesis, functionalization and deposition strategies has opened up pathways to apply and tune the inherent sensor potential of QD materials. Currently four main mechanisms are employed that utilize the highly sensitive nature of a tailored QD bandgap, including Förster Resonance Energy Transfer (FRET), Bioluminescent Resonant Energy Transfer (BRET) [77], Charge Transfer [78], and chemiluminescence [79].

By far the most common route to create QD sensor platforms utilize the phenomena of fluorescence resonance energy transfer (FRET), also referred to as Förster resonance energy transfer. Commonly used in bioimaging to serve as a molecular-level ruler, allowing Angstom-level resolution [80,81], it has proven to be a versatile mechanism to harness the QDs quantized bandgap properties for multiple sensor designs. The basic FRET principles require an overlapping donor emission and acceptor absorbance energy bands. Serving as an energy donor, energy from the excited-state QD can be transferred non-radiatively to the acceptor molecule. Therefore some of the energy that would have been given off by the donor molecule through electromagnetic radiation is now transferred to the acceptor molecule by resonance through the dipole moments of the two molecules. The degree of FRET seen in a specific donor/acceptor pair is directly related to their separation distance, donor emission and acceptor absorbance spectral overlap, and orientation of the donor/acceptor dipole moments. Calculation of the FRET efficiency between a donor and acceptor requires the determination of the most telling parameter, the Förster radius; which is defined as the distance between the donor and acceptor where 50% of the donor's energy is transferred via FRET. The Förster distance (R_0_) is defined as:

(1.1) $${{\text{R}}_{0}}^{6}=\;\left(8.8\times 10^{23}\right)\;\left(\kappa^{2}\right)\;\left({\eta_{\text{D}}}^{4}\right)\;\left(\Phi_{\text{D}}\right)\text{J}\;\left(\lambda \right)$$

where κ is the dipole orientation factor, Φ_D_ is the quantum yield of the donor, J is the normalized overlap integral between the donor and acceptor, and η is the refractive index of the medium. After the Förster radius is determined, the FRET efficiency of the donor/acceptor pair at various separation distances can be determined; a value which indicates the percentage of non-photonic emission from the donor, which is also the percentage of energy transferred to the acceptor through electron resonance. FRET efficiency (E) can be calculated using the equation:

(1.2) $$\text{E}\;=\;\left({{\text{R}}_{0}}^{6}\right)/\left({{\text{R}}_{0}}^{6}+\;{\text{R}}^{6}\right)$$

Numerous optical detection techniques have been created to translate the high sensitivity provided by FRET in sensor applications [82–88]. Utilizing the QD as the energy donor, sensing mechanisms have been achieved through modulation in separation distance, or presence of, a FRET coupled acceptor. A typical QD-biomolecular FRET coupled sensor is activated through the association or disassociation of the energy coupled FRET acceptor at the QD surface. The association of the acceptor within FRET coupling proximity, typically less than 10 nm, facilitates coupling and can be directly monitored thru alterations in the QD optical emission [84]. QD-biomolecule FRET has been successfully used to develop and demonstrate QD-based biomolecular detection systems [68,69,89]. Additional incorporation of FRET tethers [82] and non-traditional FRET approahes [83] have opened up unique *in vivo* sensor/imaging [85], drug delivery [86], automated chemical analysis review [90], nanomedicine [91,92], and nucleic acid diagnostics [93].

Although not as well-known as FRET, a new sensing mechanism known as bioluminescent resonant energy transfer has emerged [94–96]. Rather than achieving the excited donor state through electromagnetic excitation, the BRET mechanism relies on achieving electron excitation through bio-chemical reactions. Following electron excitation, the same mechanism for electron relaxation occurs as electromagnetic radiation emission seen in FRET. Utilizing the BRET approach, new multiplexed sensor platforms specifically tailored to reactive biomolecular species have been developed [77].

### 3.3. Quantum Dot—Optical Protein Coupled Sensors

**Figure 3.**
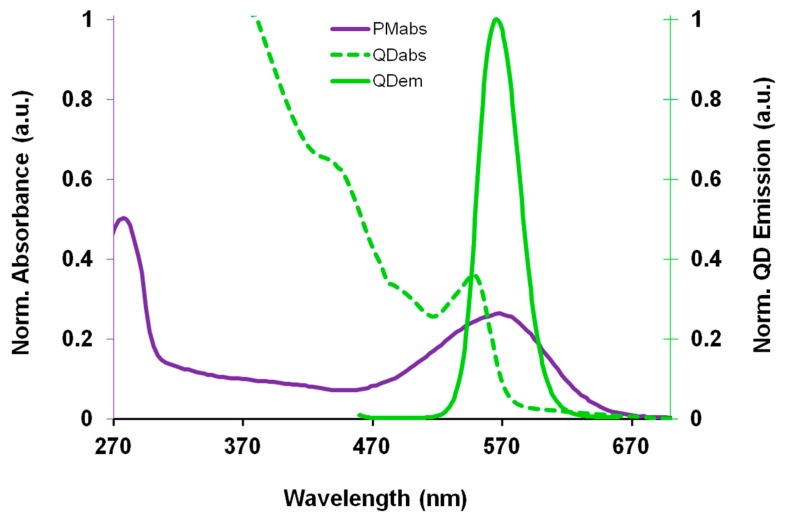
FRET coupling energy bands of bR (purple solid) and size-selected CdSe/ZnS QDs (green dashed), with overlapping QD emission (green solid).

A new approach to QD sensors is to utilize the FRET coupling mechanism to not only detect the presence of the energy acceptor molecule, but for the energy acceptor to utilize the donated QD energy to enhance its innate biomolecular function. This biomolecular enhancement has been demonstrated in QD-bacteriorhodopsin FRET coupled hybrid systems. While the application of bR can be hindered due to its relatively limited spectral activation range in the visible spectrum, coupling bR to inorganic QDs capable of capturing a broader spectral range and transferring the captured energy directly to the bR retinal. Recent studies have shown that bR molecules and colloidal QDs together have the ability to participate in FRET coupling [97] and has been optimized for maximal FRET energy conversion [97–99]. The spectra displayed in Figure 3 demonstrates the spectra overlap of the 1st QD absorption peak, occurring at 544 nm, with the bR_570 nm_ absorption peak; which is the optimal region for FRET coupling.

**Figure 4.**
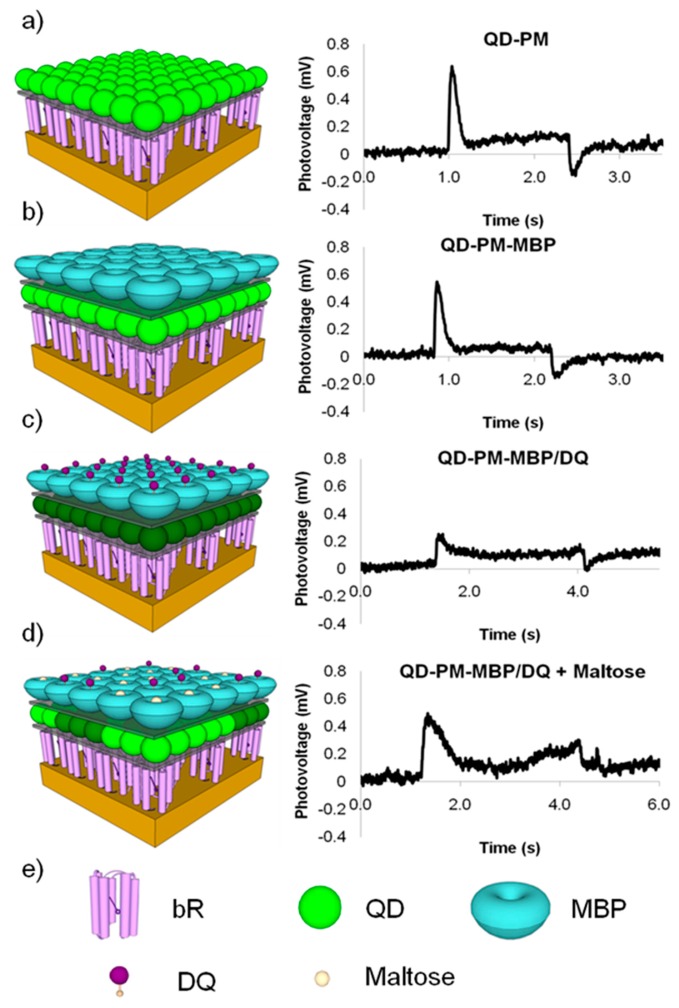
QD-bR hybrid sensor with (**a**) base bR-QD bioelectronic platform output; (**b**) output following the addition of a MBP layer to create the bR-QD-MBP electrode; (**c**) dropping the bR's photoelectric output upon dark quencher binding; and (**d**) restoration of the bR photovoltage to near original magnitudes; (**e**) Legend for materials.

Bioconjugation schemes including zero-length EDC linkers and biotin-streptavidin complexes have been employed to create QD-bR hybrid materials. To utilize bR's optoelectronic properties, which are dominantly powered by the QD energy coupling in this hybrid system, the aforementioned LBL deposition methodology has been employed to create a nanoscale hybrid QD-bR electrode system. Utilizing the maltose binding protein-dark quencher sensor methodology developed by Medintz *et al.*, a maltose sensing prototype system was developed for the QD-bR system where the sensor is monitored directly by changes in bR optocelectronic response. As shown in Figure 4, this sensor system is highly sensitive to target detection and can be analyzed in near real-time utilizing simple electronic platforms.

As shown in the bR photocurrent output in Figure 4, the formation of a QD-dark quencher FRET pair diminishes the amount of QD energy available for the bR photocycle, ultimately reducing the bR photocurrent output by over 50%. Addition of the target maltose molecule competitively displaces the dark quencher molecule from the MBP binding pocket, largely restoring the QD-bR energy transfer relationship. In the prototype sensing system, the detection of maltose was signaled by the instantaneous increase in the bR photocurrent output, reaching near original magnitudes prior to dark quencher addition. The biosensing prototype operates when the energy transfer relationship is altered upon target binding, demonstrating the applicability of a QD-bR hybrid system for sensor applications. The electrical nature of this sensing substrate will allow for its efficient integration into a nanoelectronics array form, potentially leading to a small-low power arrayed-sensing platform.

## 4. Noble Metal Nanoclusters

Nanomaterials have been an extremely popular research area in the past decades and have found applications in a wide variety of fields due to their size-dependent mechanical [100–102], electrical [103,104], and optical [105,106] properties that are not possible in bulk materials. In the biology and biomedical field, nanomaterials and nanostructures have been intensely researched, particularly for their size dependent fluorescent properties, which can be utilized for bioimaging and sensing applications [107]. Semiconductor quantum dots (QD) [108–111] and noble metal nanoparticles (NP) [112–115] have been the subject of intense research as a replacement to small molecule dyes and fluorescent proteins due to their excellent photostability and fluorescence tunability. However, the large size of NPs and the toxicity of the heavy metals used to create QDs limit their biocompatibility, making them unsuitable for *in vivo* applications [116].

Noble-metal nanoclusters (NCs) typically have sizes smaller than 2 nm and have been extensively researched as a new type of fluorophore [117–121]. Unlike NPs, whose size is comparable with the electron mean-free path (e.g., 20 nm for Au) and whose absorbance is based on surface plasmon resonance [112], noble metal NCs have sizes comparable with the Fermi wavelength (∼0.5 nm for Au and Ag), and exhibit fluorescence based on discrete electronic states [122]. Due to their small size and molecular-like properties, noble metal NCs are typically regarded as the “missing link” between atomic and nanoparticle behavior [123]. The fluorescent properties of noble metal NCs are very similar to those of the semiconductor QDs previously discussed, however, unlike QDs noble metal NCs are extremely biocompatible, making them suitable for *in vivo* applications. This greatly improves the potential biolabeling and biosensing applications of noble metal NCs over their semiconductor QD counterparts.

Due to their extremely small size, high quantum yield (QY), and excellent biocompatibility and photostability [124], NCs have received a large amount of attention in the biomedical fields for bioimaging and sensing applications [120,122,125]. Further, since the fluorescence of NCs is based on discrete electronic states, they exhibit a size-dependent fluorescence based on the number of atoms included in the cluster. This fluorescence stems from the energy difference between the highest occupied molecular orbital (HOMO) and the lowest unoccupied molecular orbital (LUMO) and the emission is caused by the transition between the sp-band (excited state) and the d-band (ground state).

### 4.1. Nanocluster Synthesis

The photoluminescence of noble metals was first reported over forty years ago by Mooradian [125], however, the extremely low quantum yield (QY) on the order of 10^−10^ severely limited the practical use of this observed phenomenon. However, spurred by the interesting biomedical applications of NPs and quantum dots, highly reproducible synthesis techniques have been developed for water-soluble noble metal NCs with quantum yields on the order of 10^−2^−10^−1^, making them practical for fluorescence based biolabeling and sensing applications.

Noble metal nanoclusters generally consist from a few to a hundred atoms with sizes comparable to the Fermi wavelength [123]. Although Au [126–137] and Ag [138–143] are the most widely used metals in NC synthesis, synthesis methods have also been developed for Cu [144] and Pt [145,146]. In order to protect from aggregation, these atoms are bound to a protective ligand. Protective ligands include proteins [132], DNA [140], dendrimers [130], polymers [142], and thiols [126]. Aside from protecting against aggregation, the choice of ligand also influences the geometry and size of the NCs, allowing for tunable fluorescence properties.

NC synthesis methods generally fall into two categories: the top-down and bottom-up approach [147]. The top-down approach generally involves etching a relatively large nanoparticle into smaller NCs while the bottom-up approach involves a chemical reaction to directly build up metal atoms on a ligand template. Key factors that influence the synthesis of high quality NCs are a strong interaction between the ligand and the metal ions, strict reducing conditions, and a relatively long aging time [124].

#### 4.1.1. Au Nanocluster Synthesis

One of the first major breakthroughs in the synthesis of nanoclusters was thiolate-protected Au NCs [126–129]. In thiolate-based NC synthesis, a thiol ligand (such as glutathione) forms a monolayer on the surface of a metal atom cluster, this subset of NCs are generally referred to as monolayer-protected clusters (MPCs) [121]. In 1998, Whetten *et al.* [126] developed a synthesis method to create 28-atom Au clusters protected by a glutathione (GSH) monolayer (Au_28_(SG)_16_). The synthesized clusters exhibited an emission at 800 nm (when excited at 500 nm) with a quantum yield of (3.5 ± 1) × 10^−3^ [129]. Following this research, Tsukuda *et al.* [128] discovered that synthesis of GSH-protected Au NCs actually produces multiple cluster sizes (termed “magic numbers”) which can be separated using polyacrylamide gel electrophoresis (PAGE). Using mass spectrometry, clusters of 18-, 21-, 25-, 28-, 32-, and 39- atoms were discovered and isolated.

In 2003, Zheng *et al.* [130] developed a synthesis method to produce water-soluble 8-atom Au nanoclusters stabilized by a poly(amidoamine) (PAMAM) dendrimer. In order to prepare these NCs, 0.5 μmol of G4-OH and 1.5 μmol of HAuCl_4_*nH_2_O were dissolved in 2 mL of distilled water, NaBH_4_ was added as a reduction agent. This solution was then stirred for 2 days until the reaction was complete. This reaction resulted in both Au NCs as well as larger NPs, which were removed by centrifugation. These Au_8_ NCs exhibited a fluorescence emission peak at 450 nm upon excitation at 384 nm and had an impressive QY of around 41%. In a later paper Zheng *et al.* [131] reported that the size of these NCs could be tuned by altering the Au:PAMAM concentration ratio. Using this method, Au_5_, Au_8_, Au_13_, Au_23_, and Au_31_ NCs were created. It was also discovered that as the size of the NCs increased, the fluorescence emission peak exhibited a red-shift, allowing for a tunable fluorescence peak from UV to near IR. However, larger NCs also resulted in lower QY, as shown in Table 2.

**Figure 5.**
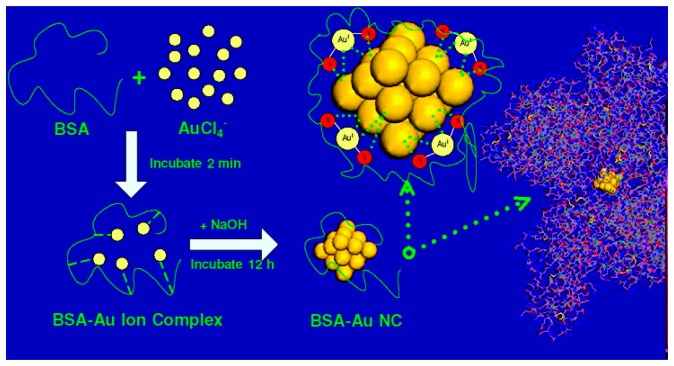
Formation of BSA-AuNCs [132]. Reprinted with permission from [132]. Copyright (2014) American Chemical Society.

In 2008, Xie *et al.* [132] developed a one-pot, “green” technique to synthesize Au_25_ NCs using the common protein bovine serum albumin (BSA) as the protective ligand (Figure 5). The synthesis method involved adding 5 mL of aqueous HAuCl_4_ solution (10 mM, 37 °C) to 5 mL of BSA solution (50 mg/mL, 37 °C), stirring vigorously for 2 min, and adding 0.5 mL of NaOH solution (1 M) to bring the pH of the solution to ∼12. The solution was then incubated at 37 °C for ∼12 h. This method produces Au_25_ NCs with a fluorescence emission peak at 640 nm with a UV-excitation (365 nm) and a QY of ∼6%. Although the QY of this method is somewhat low compared to other methods, the simple, economical recipe, low synthesis time, large Stokes shift, and biocompatibility of this method have made it a very popular method for creating fluorescent NCs for bioimaging and sensing applications.

In 2009, Cheng-An *et al.* [137] developed a top-down method to synthesize Au NCs protected by dihydrolipoic acid (DHLA) by etching larger NPs. The base NPs used in this synthesis method were 6 nm diameter Au NPs (stabilized with didodecyldimethylammonium bromide (DDAB). These NPs were dissolved in a toluene solution, bringing the average diameter to approximately 5.5 nm. Adding a gold precursor (AuCl_3_ or HAuCl_4_) to the DDAB-toluene solution further dissolved the NPs to an average size of around 3 nm, which completely removed the surface plasmon absorption, resulting in NC behavior. In order to make the resulting NCs water-soluble for biological applications, the NCs were subjected to a ligand exchange with DHLA by adding a DHLA:TBAB solution to the AuNC:toluene solution and performing a centrifugation on the resulting mixture to isolate the AuNCs. The resulting AuNC@DHLA particles have an average diameter of around 1.5 nm with an emission peak at 650 nm (with excitation at 490 nm) and a QY of around 3.45% in methanol and 1.8% in water.

#### 4.1.2. Ag Nanocluster Synthesis

In 2002, following in the footsteps of water-soluble gold nanoclusters, Dickson *et al.* [148] attempted to create Ag NCs using a PAMAM dendrimer as a stabilizing agent. Although synthesis of Ag NCs (Ag_2_ and Ag_8_) was demonstrated prior in 2001 through photoactivation of silver oxide thin-films [149], little control of these surface bound nanoclusters was possible [148]. PAMAM encapsulated Ag NCs were created by dissolving 0.5 μmol of G4-OH and 1.5 μmol of AgNO_3_ into 1 mL of distilled water and adjusting to neutrality with acetic acid. Although this process was successful in creating Ag_2_ and Ag_8_ NCs, they were very prone to aggregation in high concentrations or introduction to a buffer [138], resulting in the production of large Ag nanoparticles.

**Figure 6.**
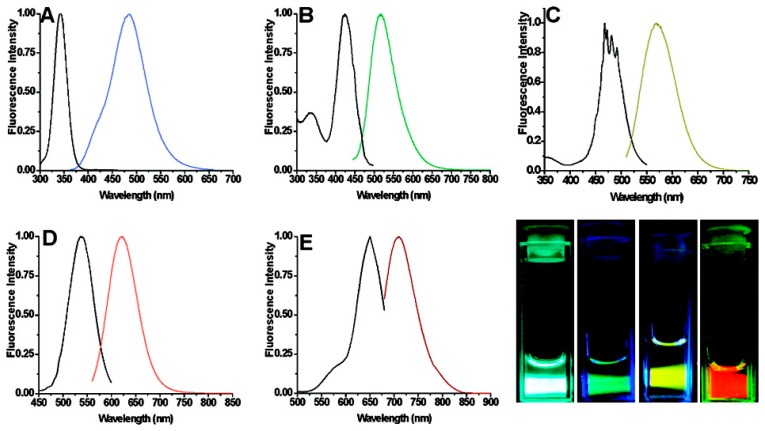
Excitation and emission spectra for ss-DNA encapsulated AgNCs. (**A**) Blue Emitters (5′-CCCTTTAACCCC-3′) (**B**) Green Emitters (5′-CCCTCTTAACCC-3′) (**C**) Yellow Emitters (5′-CCCTTAATCCCC-3′) (**D**) Red Emitters (5′-CCTCCTTCCTCC-3′) (**E**) near-IR emitters (5′-CCCTAACTCCCC-3′) (**F**) Images of solutions A–D [140]. Reprinted with permission from [140]. Copyright 2014 American Chemical Society.

In 2004, Dickson *et al.* [139] developed a synthesis technique to create Ag NCs using a DNA template. This synthesis technique involved combining AgNO_3_ and the 12-base oligonucleotide 5′-AGGTCGCCGCCC-3′, cooling the solution to 0 °C, and adding NaBH_4_ and vigorously shaking. Though mass spectral analysis, it was determined that this synthesis method produced Ag NCs in the range of 1–4 atoms. In 2008, Richards *et al.* [140] expanded on the idea of synthesizing Ag NCs using single-stranded DNA (ssDNA) as a template by exploring the effect of using different DNA sequences. The recipe consisted of adding a 6:1 molar ratio of AgNO_3_ to 50 μM of an oligonucleotide solution, the mixture was then reduced with NaBH_4_ after 15 min. It was found that the fluorescence emission could be tuned by altering the sequence of the DNA template. Blue emitters (λ_em_ = 485 nm) were created using a 5′-CCCTTTAACCCC-3′ template, green emitters (λ_em_ = 520 nm) were created using 5′-CCCTCTTAACCC-3′, yellow emitters (λ_em_ = 572 nm) were created using 5′-CCCTTAATCCCC-3′, red emitters (λ_em_ = 620 nm) were created using 5′-CCTCCTTCCTCC-3′, and near-IR (NIR) emitters (λ_em_ = 705 nm) were created using 5′-CCCTAACTCCCC-3′ (Figure 6). Although the yellow, red, and NIR emitters showed great photostability, the blue and green emitters did not, making them unsuitable for practical use. The QY of the yellow, red, and NIR emitters were also excellent (over 30%), as can be seen in Table 3.

#### 4.1.3. Cu and Pt Nanocluster Synthesis

Although the synthesis of Cu NCs is relatively scarce due to susceptibility to oxidation and difficulty of preparation [124], synthesis methods for Cu NCs using a variety of ligands have been developed. In 1998, Tomalia *et al.* [150] and Crooks *et al.* [151] simultaneously developed a synthesis method to create Cu NCs using a PAMAM dendrimer template. In 2011, Pradeep *et al.* [144] synthesized Cu NCs using a BSA template. These clusters were composed of 5- and 13-atom cores and had an emission peak at 410 nm (with an excitation at 325 nm), with a QY of 0.15. Synthesis methods for Cu NCs using dsDNA [152] and ssDNA [153] have also recently been developed.

Synthesis strategies have also been developed for the creation of Pt NCs. In 2011, Tanaka *et al.* [145] created blue-emitting Pt NCs using a PAMAM dendrimer template and a NaBH_4_ reductant. These NCs were composed of five Pt atoms and showed and emission peak at 470 nm, with a QY of 18%. In 2013, Tanaka *et al.* [146] developed a synthesis method to create a green version of the Pt NCs while still using a PAMAM dendrimer template by using a milder trisodium citrate reductant. These clusters consist of 8 Pt atoms (as opposed to the five atoms contained in the blue emitting version) and had an emission peak at 520 nm (with a 460 nm excitation) with a QY of 28%.

### 4.2. Applications of Noble Metal NCs

The unique fluorescent properties of NCs combined with the fact that they can be templated with a wide variety of ligands have made noble metal nanoclusters an excellent candidate for a wide variety of sensing and biolabeling applications. This section will provide a brief overview of a few of the recent advances in the application of noble metal NCs.

#### 4.2.1. Heavy Metal Sensing

The environmental and health concerns associated with the presence of heavy metal ions has pushed the development of efficient, accurate, and economical sensing. The properties of noble metal NCs have spurred an intense research push into the creation of sensors for heavy metals in environmental and biological samples [154–163].

In 2007, Huang *et al.* [154] introduced a method of sensing mercuric ions (Hg^2+^) using 11-mercaptoundecanoic acid (11-MUA)-protected gold nanoclusters (11-MUA AuNCs). Since the presence of Hg^2+^ caused aggregation of the 11-MUA AuNCs, it was found that the concentration of Hg^2+^ could be determined by measuring the amount of aggregation induced fluorescence quenching had occurred. The emission peak of the 11-MUA AuNCs was located at 530 nm (with excitation at 375 nm) with a QY of approximately 3.1%. It was determined that the decrease in fluorescence had a logarithmic relation with the concentration of Hg^2+^ over the concentration range of 10 nM to 10 μM, with an LOD of 5nM, which is approximately half the maximum concentration allowed in drinking water by the Environmental Protection Agency (EPA).

In 2010, Xie *et al.* [155] developed a method for the detection of Hg^2+^ using BSA-protected gold nanoclusters (BSA-AuNCs). It was found that in the presence of Hg^2+^, the emission peak of BSA-AuNCs at 640 nm (excitation wavelength of 470 nm) could be quenched within seconds due to the high-affinity metallophilic Hg^2+^-Au^+^ interactions. It was discovered that there is a linear relationship between the fluorescence quenching and Hg^2+^ concentration over a concentration range of 1–20 nM with a LOD of 0.5 nM, which is approximately 20 times lower than the maximum allowable concentration of mercury in drinking water permitted by the Environmental Protection Agency (EPA). A similar method of sensing Hg^2+^ using trypsin-stabilized AuNCs was developed in 2011 by Kawasaki *et al.* [157], although the LOD of this method was relatively high (50 ± 10 nM), the detectable concentration range was much higher (50–600 nM). An extension of this method was employed by Dai *et al.* [156] to detect melamine concentrations. It was found that the fluorescence quenching behavior of Hg^2+^ on BSA-AuNCs could be reversed by the introduction of melamine. When a known Hg^2+^ concentration is mixed with a melamine concentration before being added to the BSA-AuNC solution, the “anti-quenching” ability of the melamine can be detected and shows linear behavior in the concentration range of 0.5–10 μM, with a LOD of 0.15 μM.

In 2008, Dong *et al.* [159] developed a method to detect copper ions (Cu^2+^) using PMAA-templated Ag NCs (PMAA AgNC). It was found that Cu^2+^ quenched the fluorescence of the PMAA AgNC by binding with the free carboxylic groups of the PMAA polymers. A linear relationship was found between the quenching of the emission peak of the PMAA AgNCs at 615 nm (excitation at 510 nm) and the concentration of Cu^2+^ in the concentration range of 10 nM to 6 μM, with a detection limit of 8 nM.

In 2013, Zhang *et al.* [158] developed a sensor to detect Cu^2+^ in water and biological samples using glutathione-protected gold nanoclusters (GS-Au NCs). It was found that the fluorescence emission peak of the GS-Au nanoclusters at 720 nm (with an excitation wavelength of 420 nm) was quenched in the presence of Cu^2+^ ions. This behavior was found to be caused by the complexation between the copper ions and GS-ligands of the GS-Au nanoclusters. A linear relationship was found between fluorescence quenching and Cu^2+^ concentration in the range of 1.00 × 10^−7^ to 6.25 × 10^−6^ mol/L, with a detection limit of 86 nM. After performing this test on 17 different metal ions, it was found that fluorescence quenching of the GS-Au NCs was caused by Cu^2+^, Hg^2+^, and Pb^2+^. With the addition of an EDTA solution, it was found that the fluorescence could be restored if the quenching was caused by Cu^2+^ or Pb^2+^, but would stay constant if the quenching was caused by Hg^2+^, and that quenching caused by Pb^2+^ created a turbid solution, which would become clear with increasing concentrations of EDTA, increasing the selectivity of this sensing method.

In 2011, Chang *et al.* [160] developed a method of sensing sulfide ions (S^2−^) using DNA-templated gold/silver nanoclusters (DNA-Au/Ag NCs). Sensing was based on the fluorescence quenching by changing of the template DNA from hairpin to random coil structures as a result of the interaction between S^2−^ and the gold/silver ions. A linear relation between the quenching of the emission peak at 630 nm and the concentration of S^2−^ ions in the concentration range of 10 nM and 9 μM, with a detection limit of around 0.83 nM. In 2013, Cui *et al.* [161] developed a sensor for the detection of S^2−^ using Bovine Serum Albumin protected gold nanoclusters (BSA-AuNC). It was found that in the presence of S^2−^, the fluorescence emission peak at 635 nm (with an excitation wavelength of 489 nm) of the BSA-Au NCs was quenched due to degradation of the structure caused by the S^2−^ ions. A linear relationship between the fluorescence quenching and S^2−^ concentration was found in the range of 0.1–30 μM with a LOD of 0.029 μM. It was also found that the presence of a multitude of other metallic ions and anions exhibited a negligible amount of fluorescence quenching on the BSA-Au NCs.

**Figure 7.**
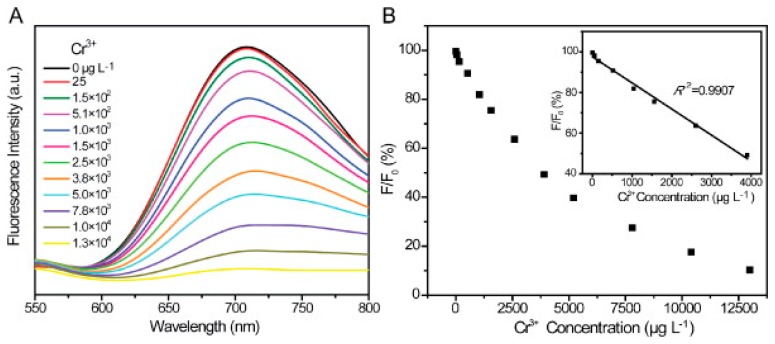
(**A**) Effect of Cr(III) concentration on fluorescence of GSH-AuNCs (pH = 6.5) (**B**) Relative fluorescence with increasing Cr(III) concentration [162]. Reprinted from [162], with permission from Elsevier.

**Figure 8.**
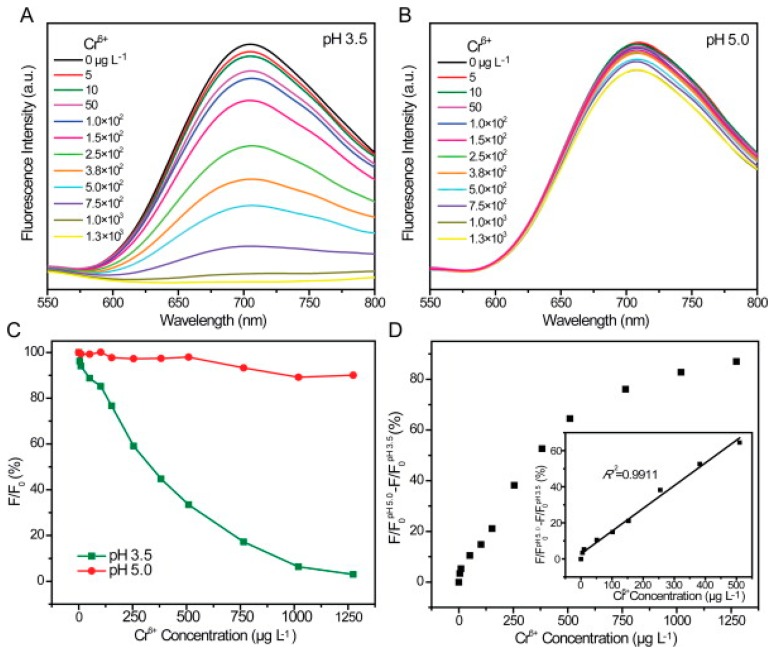
(**A**) Effect of Cr(VI) concentration on fluorescence of GSH-AuNCs (pH = 3.5) and (**B**) pH = 5.0 (**C**) Relative fluorescence of GSH-AuNCs with increasing Cr(VI) concentration (**D**) Difference in relative fluorescence between pH = 3.5 and pH = 5.0 solutions with increasing Cr(VI) concentration [162]. Reprinted from [162], with permission from Elsevier.

In 2013, Zhang *et al.* [162] developed a method of selectively detecting chromium (III) and chromium (VI) in water samples using glutathione-stabilized gold nanoclusters (GSH-Au NCs). It was found that the presence of Cr (III) and Cr (VI) caused quenching of the emission peak of the GSH-Au NCs at 710 nm (with an excitation wavelength of 410 nm), it was also found that the detection of Cr (III) and Cr (VI) could be separated based on the pH of the solution. It was found that at a pH of 6.5, the fluorescence quenching of GSH-Au NCs increased linearly with increasing Cr(III) concentration in the range of 2 5–3800 μg/L, with complete quenching occurring at 13 mg/L and a limit of detection of 2.5 μg/L (Figure 7). Further, at a pH of 6.5, Cr(VI) exhibited negligible fluorescence quenching. When the pH of the solution was lowered to 3.5–5, the fluorescence quenching of the GSH-Au NCs was completely dependent on the concentration of Cr(VI), with Cr(III) having a negligible impact. The quenching behavior had a linear relationship with the concentration of Cr(6) in the range of 5–500 μg/L with a limit of detection of 0.5 μg/L (Figure 8).

In 2013, Mu *et al.* [163] developed a method of detecting Ferric Iron (Fe^3+^) using L-proline stabilized gold nanoclusters. It was found that the presence of Fe^3+^ caused a fluorescence quenching at the emission peak of 440 nm (with an excitation frequency of 365 nm) due to aggregation of the Au NCs in the presence of ferric iron, decreasing the size dependent fluorescence intensity of the nanoclusters.

A linear relationship between the fluorescence quenching and Fe^3+^ concentration was observed in the range of 5–2000 μM with an LOD of 2 μM. This sensing method can be completed in under 3 min, making it much quicker and economical than other methods of ferric iron detection.

#### 4.2.2. Molecule and Protein Sensing

*In-vitro* sensing of molecules and proteins has been a very popular research topic in the past decades. The properties of noble metal NCs have made them a popular candidate for the sensitive and selective detection of molecules and proteins [164–174].

Liu *et al.* [164] developed a method of detecting cyanide (CN^−^) concentration in aqueous solution using BSA protected gold nanoclusters (BSA-AuNCs). It was found that at a pH value of 12.0, the presence of cyanide causes a fluorescence quenching at the emission peak of 460 nm (excitation wavelength of 365 nm) of the BSA-AuNCs due to etching of the gold atoms from the CN^−^. It was found that there was a linear relationship between the fluorescence quenching and CN^−^ concentration over a range of 200 nM–9.5 μM, with a LOD of 200 nM, which is 14 times lower than the maximum allowed concentration of CN^−^ in drinking water permitted by the World Health Organization. It was also found that at the pH level used in this analysis, the detection of cyanide concentration was not affected by the presence of other common ions and anions, demonstrating great selectivity over other metals found in water.

In 2011, Martinez *et al.* [165] developed a method of using DNA aptamer-templated AgNCs to detect specific proteins. This group theorized that by synthesizing AgNCs directly to a recognition ligand, a multitude of different proteins could be detected. In order to test this hypothesis, the group synthesized AgNCs on a cytosine rich thrombine APT29 DNA sequence in order to detect human α-thrombin. It was found that upon addition of thrombin protein, the fluorescence of the aptamer-AgNCs was significantly quenched. The detection limit was found to be 1 nM, and saturation of the quenching effect occurred at approximately 1000 nM. It was also found that addition of denatured thrombin, thrombin prebound to an aptamer, and a number of different proteins (*i.e.*, BSA, streptavidin, and platelet derived growth factor (PDGF)) had no effect of the fluorescence of the aptamer-AgNCs, showing a high selectivity of the AgNC sensor to the target protein. This aptamer method of detection was also used in 2011 by Zhou *et al.* [173] to detect cocaine. The method employed by this group was a “turn on” method utilizing AgNCs templated with G-rich CocaS1 and CocaS2 aptamers. It was found that with no cocaine present, the AgNCs created by these two aptamer exhibited a weak fluorescence. However, when cocaine is present, it is bound by the two g-rich aptamers, whose proximity results in the creation of AgNCs with a greatly enhanced fluorescence. The detection limit of this method was found to be 0.1 μM and a logarithmic relationship was found between fluorescence enhancement and cocaine concentration in the range of 0.5 μM–1 mM.

Wen *et al.* [166] developed a highly sensitive and selective sensor based on polyethylene-capped silver nanoclusters (PEI-Ag) to simultaneously detect hydrogen-peroxide (H_2_O_2_) and glucose. H_2_O_2_ is sensed by its ability to quench the fluorescence of the PEI-Ag nanoclusters, which was found to be caused by the oxidation of the Ag nanoclusters in the presence of H_2_O_2_. In the presence of H_2_O_2_, The emission peak of PEI-Ag NCs at 455 nm (excitation peak = 375 nm) was found to exhibit quenching within 30 min with a linear response over a concentration range of 500 nM–100 μM with a detection limit of 400 nM. It was also found that when exposed to a glucose oxidase (GOx), glucose can be oxidized in the presence of O_2_ and H_2_O to create H_2_O_2_ and gluconic acid. By utilizing the quenching effect of H_2_O_2_, the PEI-Ag nanoclusters can be used to detect glucose concentration. It was found that the fluorescence quenching of PEI-Ag NCs exhibited a linear relationship with glucose concentration over the range of 1–1000 μM, with a detection limit of 800 nM. It was also found that when using the glucose oxidase, the fluorescence quenching of the PEI-Ag NCs was not affected by the presence of other carbohydrates or metal ions, leading to a very selective sensor. Further, since a multitude of O_2_-dependent oxidase enzymes can produce H_2_O_2_, this sensing method has the potential to be employed to sense a multitude of materials.

In 2012, Wang *et al.* [167] developed a method of detecting a multitude of amino acids using BSA AuNCs that were modulated with different metal ions. This detection method utilized the ratiometric fluorescent responses of the two peaks of the BSA AuNCs (blue at 425 nm from the BSA and red at 635 nm from the Au NCs) to the interaction of amino acids with the bound metal NCs. It was found that the ratio of the blue and red peaks were influenced by the addition of metal ions (e.g., Ni^2+^, Pb^2+^, Cd^2+^, Zn^2+^) as well as the interaction between amino acids and the added metal ions. By using multiple BSA AuNC probes modulated by different metal ions, a variety of amino acids can be detected.

In 2013, Dou *et al.* [170] developed a method of detecting deoxyribonuclease I (DNase I) using DNA-templated gold/silver nanoclusters (DNA-Au/Ag NCs). The method of detection is based on the fluorescence quenching of the DNA templated NCs due to the digestion of the DNA (5′-CCCTTAATCCCC-3′) template by DNase I. It was found that in the presence of DNase I, the fluorescence peak of the NCs at 560 nm (with excitation wavelength 370 nm). It was found that there is a linear relationship between fluorescence quenching and DNase I concentration between 0.013 and 60 μg/mL with a LOD of 3 ng/mL. It was also found that the detection of DNase I was not affected by the presence of other enzymes.

Noble metal nanoclusters are quickly becoming a very promising new class of fluorophores due to their extremely small size, biocompatibility, and tunable fluorescent properties. Recent advances on the synthesis of NCs have also made them increasingly effective and economical. The unique properties of NCs have also spurred research into the application of NCs for a wide variety of sensing and biolabeling applications.

In addition to the applications mentioned, other novel properties of noble metal NCs are constantly being discovered. In 2007, Brown *et al.* [175] developed a method of sensing hydrogen using the tunneling between Pd clusters deposited in a thin film on a pair of contacts. In 2012, Zhang *et al.* [176] discovered that the fluorescent intensity of BSA AuNCs was sensitive to pressure due to conformational changes of the ligand. They also found that there was a linear relationship between applied pressure and fluorescent intensity, indicating that noble metal NCs may have the potential to sense mechanical phenomena. In 2005, Qiang *et al.* [177] synthesized Fe NCs in a thin film which exhibited exceptional magnetic properties. The high magnetic moments exhibited by these NCs could be used to greatly enhance the contrast of MRI, as well as improve the effectiveness of cell separation and drug delivery.

The rapidly improving synthesis techniques and impressive array of potential applications of noble metal NCs make them a very popular research topic. However, although a great deal of progress has been made, a fundamental understanding of noble metal NCs is still lacking and a number of challenges in the application of nanoclusters will need to be overcome. Overall, noble metal NCs have the potential to find very interesting and potentially lucrative applications in a wide variety of fields.

## 5. Conclusions/Outlook

This article provided a brief review of recent advances in molecular sensors and electronics. optoelectric bateriorhodopsin, semiconductor quantum dots and noble metal nanoclusters are very active and exciting areas in the field of bionanotechnology, with new progress constantly being made in adapting these technologies in the creation of new biosensors and bioelectronics. It should be noted that this review is in no way a comprehensive review of the state of molecular sensors and electronics, however, the brief summary of the three technologies discussed in this article will hopefully be sufficient to introduce the reader to the relatively new and fruitful field of bionanotechnology and inspire them to engage in their own research.

## Figures and Tables

**Table 1.** Reagents required to prepare *H. salinarum* growth medium [5].

**Reagent**	**Concentration**	**Per 1 Liter**
NaCl	4.28 M	250 g
MgSO_4_ (anhydrous)	81.1 mM	9.77 g
KCl	26.8 mM	2 g
NH_4_Cl	93.5 mM	5 g
Sodium citrate, 2H_2_O	10.2 mM	3 g
Glycerol	137 mM	1 mL
KH_2_PO_4_	0.735 mM	0.1 g
CaCl_2_ (anhydrous)	1.4 mM	0.2 g
Bacteriological peptone	--	10 g

**Table 2.** Measured PAMAM templated Au-NC photophysical properties [131].

**Gold**	**Excitation**	**Emission**	**QY**	**Lifetime**
Cluster	(FWHM) (eV)	(FWHM) (eV)	(%)	(ns)
Au_5_	3.76 (0.42)	3.22 (0.45)	70	3.5
Au_8_	3.22 (0.54)	2.72 (0.55)	42	7.5
Au_13_	2.86 (0.38)	2.43 (0.41)	25	5.2
Au_23_	1.85 (0.21)	1.65 (0.26)	15	3.6
Au_31_	1.62 (0.20)	1.41 (0.10)	10	

**Table 3.** Photophysical parameters of DNA-templated Ag nanoclusters [140]. Reprinted with permission from [140]. Copyright 2014 American Chemical Society.

**Species**	**Emission (nm)**	**Lifetime (ns)**	**Φ (%)**	**ε (10^5^ M^−1^ cm^−1^)**
blue	485	2.98 ± 0.01		
green	520	0.22 ± 0.01	16 ± 3	
yellow	572	4.35 ± 0.01	38 ± 2	2.0 ± 0.4
red	620	2.23 ± 0.01	32 ± 4	1.2 ± 0.3
NIR	705	3.46 ± 0.01	34 ± 5	3.5 ± 0.7

## References

[1] Haupts U., Tittor J., Oesterhelt D. (1999). Closing in on bacteriorhodopsin: Progress in understanding the molecule. Annu. Rev. Biophys. Biomol. Struct..

[2] Oesterhelt D., Stoeckenius W. (1971). Rhodopsin-like protein from the purple membrane of *halobacterium halobium*. Nature.

[3] Ventosa A., Oren A. (1996). Halobacterium salinarum nom. Corrig, a name to replace halobacterium salinarium (elazari-volcani) and to include halobacterium halobium and halobacterium cutirubrum. Int. J. Syst. Evolut. Microbiol..

[4] Oesterhelt D., Stoeckenius W., Fleischer S., Packer L. (1974). Isolation of the Cell Membrane of Halobacterium Halobium and Its Fractionation into Red and Purple Membrane. Methods of Enzymology.

[5] Anton C. (2008). Photolithography Based Patterning of Bacteriorhodopsin Films. Ph.D. Thesis.

[6] Jonas R., Koutalos Y., Ebrey T. (1990). Purple membrane: Surface charge density and the multiple effect of ph and cations. Photochem. Photobiol..

[7] He J.-A., Samuelson L., Li L., Kumar J., Tripathy S. (1999). Bacteriorhodopsin thin film assemblies—Immobilization, properties, and applications. Adv. Mater..

[8] Birge R. (1990). Photophysics and molecular electronic applications of the rhodopsins. Annu. Rev. Phys. Chem..

[9] Fisher K., Yanagimoto K., Stoeckenius W. (1978). Oriented adsorption of purple membrane to cationic surfaces. J. Cell Biol..

[10] He J.-A., Samuelson L., Li L., Kumar J., Tripathy S. (1997). Oriented bacteriorhodopsin/polycation multilayers by electrostatic layer-by-layer assembly. Langmuir.

[11] Derose J.A., Leblanc R.M. (1995). Scanning tunneling and atomic-force microscopy studies of langmuir-blodgett-films. Surf. Sci. Rep..

[12] Petty M. (1996). Langmuir-Blodgett Films an Introduction.

[13] Maximychev A., Kholmansky A., Levin E., Rambidi N., Chamorovsky A., Kononenko V., Erokhin V., Checulaeva L. (1992). Oriented purple membrane multilayers of halobacteria fabricated by langmuir-blodgett and electrophoretic sedimentation techniques. Adv. Mater. Opt. Electron..

[14] Hwang S.B., Korenbrot J.I., Stoeckenius W. (1977). Proton transport by bacteriorhodopsin through an interface film. J. Membr. Biol..

[15] Boucher J., Trudel E., Methot M., Desmeules P., Salesse C. (2007). Organization, structure and activity of proteins in monolayers. Colloid Surface B.

[16] Ikonen M., Sharonov A., Tkachenko N., Lemmetyinen H. (1993). The photovoltage signals of bacteriorhodopsin in langmuir-blodgett films with different molecular orientations. Adv. Mater. Opt. Electron..

[17] Choi H.G., Jung W.C., Min J.H., Lee W.H., Choi J.W. (2001). Color image detection by biomolecular photoreceptor using bacteriorhodopsin-based complex lb films. Biosens. Bioelectron..

[18] Miyasaka T., Maekawa Y., Koyama K. (1989). A novel photoreactive amphiphile of nitrophenylazide for immobilization of bioactive proteins. Thin Solid Films.

[19] Miyasaka T., Koyama K. (1993). Rectified photocurrents from purple membrane langmuir-blodgett films at the electrode-electrolyte interface. Thin Solid Films.

[20] Sugiyama Y., Inoue T., Ikematsu M., Iseki M., Sekiguchi T. (1997). Controlling the orientation of purple membrane fragments on an air/water interface by a new method of direct electric field application during purple membrane spreading. Jpn. J. Appl. Phys..

[21] Nicolini C., Erokhin V., Paddeu S., Sartore M. (1998). Towards a light-addressable bacteriorhodopsin-based transducer. Nanotechnology.

[22] Bertoncello P., Nicolini D., Paternolli C., Bavastrello V., Nicolini C. (2003). Bacteriorhodopsin-based langmuir-schaefer films for solar energy capture. IEEE Trans. Nanobiosci..

[23] Langmuir I., Schaefer V. (1938). Activities of urease and pepsin monolayers. J. Am. Chem. Soc..

[24] Weetall H.H., Druzhko A.B. (2003). Optical and electrical characteristics of bacteriorhodopsin gelatin films and tin-oxide coated electrodes. Curr. Appl. Phys..

[25] Fredericq E., Houssier C. (1973). Electric Dichromism and Electric Birefringence.

[26] Koyama K., Yamaguchi N., Miyasaka T. (1995). Molecular organization of bacterio-rhodopsin films in optoelectronic devices. Adv. Mater..

[27] Worldwide Protein Data Bank http://www.wwpdb.org/.

[28] Freitag S., LeTrong I., Klumb L., Stayton P.S., Stenkamp R.E. (1997). Structural studies of the streptavidin binding loop. Protein Sci..

[29] Samuelson L.A., Miller P., Galotti D.M., Marx K.A., Kumar J., Tripathy S.K., Kaplan D.L. (1992). The monomolecular organization of a photodynamic protein system through specific surface recognition of streptavidin by biotinylated langmuir-blodgett-films. Langmuir.

[30] Miyasaka T., Koyama K. (1991). Photoelectrochemical behavior of purple membrane langmuir-blodgett-films at the electrode-electrolyte interface. Chem. Lett..

[31] Winder E. (2010). Bacteriorhodopsin Protein Hybrids for Chemical and Biological Sensing. Ph.D. Thesis.

[32] Walczak K. (2009). Immobilizing Bacteriorhodopsin on a Single Electron Transistor. Ph.D. Thesis.

[33] Bauer L.A., Birenbaum N.S., Meyer G.J. (2004). Biological applications of high aspect ratio nanoparticles. J. Mater. Chem..

[34] Knoll W., Liley M., Piscevic D., Spinke J., Tarlov M.J. (1997). Supramolecular architectures for the functionalization of solid surfaces. Adv. Biophys..

[35] Knoll W., Zizlsperger M., Liebermann T., Arnold S., Badia A., Liley M., Piscevic D., Schmitt F.J., Spinke J. (2000). Streptavidin arrays as supramolecular architectures in surface-plasmon optical sensor formats. Colloid. Surface. A.

[36] Haronian D., Lewis A. (1992). Microfabricating bacteriorhodopsin films for imaging and computing. Appl. Phys. Lett..

[37] Falconnet D., Csucs G., Grandin H.M., Textor M. (2006). Surface engineering approaches to micropattern surfaces for cell-based assays. Biomaterials.

[38] Anton C., Walczak K., Lueking D., Friedrich C. Effects of Focused Ion Beam Machining on Oriented Bacteriorhodopsin Optical Protein Films.

[39] Libertino S., Fichera M., D'Arrigo G., la Mantia A., Ricceri D. (2003). Characterization and pattering of bacteriorhodopsin films on si-based materials. Synthetic Met..

[40] Crittenden S., Reifenberger R., Hillebrecht J., Birge R., Inerowicz D., Regnier F. (2005). Soft lithography based micron-scale electrophoretic patterning of purple membrane. J. Micromech. Microeng..

[41] Shiu P.J., Ju Y.H., Chen H.M., Lee C.K. (2013). Facile isolation of purple membrane from halobacterium salinarum via aqueous-two-phase system. Protein Expres Purif..

[42] Thavasi V., Lazarova T., Filipek S., Kolinski M., Querol E., Kumar A., Ramakrishna S., Padros E., Renugopalakrishnan V. (2009). Study on the feasibility of bacteriorhodopsin as bio-photosensitizer in excitonic solar cell: A first report. J. Nanosci. Nanotechnol..

[43] Al-Aribe K., Knopf G., Bassi A. Organic Photovoltaic Cells Based on Photoactive Bacteriorhodopsin Proteins.

[44] Yen C.W., Hayden S.C., Dreaden E.C., Szymanski P., El-Sayed M.A. (2011). Tailoring plasmonic and electrostatic field effects to maximize solar energy conversion by bacteriorhodopsin, the other natural photosynthetic system. Nano Lett..

[45] Karna S., Mallick G., Friedrich C., Griep M. (2010). Engineered Nano-Bio Hybrid Electronic Platform for Solar Energy Harvesting.

[46] Fukuzawa K. (1994). Motion-sensitive position sensor using bacteriorhodopsin. Appl. Opt..

[47] Xu J., Bhattacharya P., Varo G. A photoreceiver based on the monolithic integration of oriented bacteriorhodopsin and gaas modfet's.

[48] Bhattacharya P., Xu J.A., Varo G., Marcy D.L., Birge R.R. (2002). Monolithically integrated bacteriorhodopsin-gaas field-effect transistor photoreceiver. Opt. Lett..

[49] Xu J., Bhattacharya P., Varo G. (2004). Monolithically integrated bacteriorhodopsin/semiconductor opto-electronic integrated circuit for a bio-photoreceiver. Biosens. Bioelectron..

[50] Shin J., Bhattacharya P., Yuan H.C., Ma Z.Q., Varo G. (2007). Low-power bacteriorhodopsin-silicon *n*-channel metal-oxida field-effect transistor photoreceiver. Opt. Lett..

[51] Anton C., Walczak K., Lueking D., Friedrich C. (2010). Integration of optical protein with electronics for bio-nanosensors. J. Nanosci. Nanotechnol..

[52] Anton C., Friedrich C., Lueking D. Photolithographic patterning of bacteriorhodopsin films.

[53] Walczak K., Bergstrom P., Friedrich C. (2011). Light sensor platform based on the integration of bacteriorhodopsin with a single electron transistor. Active Passive Electron. Comp..

[54] Walczak K., Acharya M., Lueking D., Bergstrom P., Friedrich C. Integration of the bionanomaterial bacteriorhodopsin and single electron transistors.

[55] Sharkany J.P., Korposh S.O., Batori-Tarci Z.I., Trikur I.I., Ramsden J.J. (2005). Bacteriorhodopsin-based biochromic films for chemical sensors. Sens. Actuators B Chem..

[56] Korposh S.O., Sharkan Y.P., Ramsden J.J. (2008). Response of bacteriorhodopsin thin films to ammonia. Sens. Actuators B Chem..

[57] Griep M. (2008). Quantum Dot/Optical Protein Bio-Nano Hybrid System Biosensing. Ph.D. Thesis.

[58] Griep M., Lueking D., Mackay R., Karna S., Friedrich C. Hybrid protein-quantum dot nanoscale structures for biosensing and photovoltaics.

[59] Griep M., Martin J., Rodriguez V., Winder E., Lueking D., Mackay R., Friedrich C., Karna S. Multi-functional protein-qd hybrid substrates for photovoltaics and real-time biosensing.

[60] Griep M.H., Winder E.M., Lueking D.R., Friedrich C.R., Mallick G., Karna S.P. (2010). Optical protein modulation via quantum dot coupling and use of a hybrid sensor protein. J. Nanosci. Nanotechnol..

[61] Griep M., Winder E., Lueking D., Garrett G., Karna S., Friedrich C. (2012). Forster resonance energy transfer between core/shell quantum dots and bacteriorhodopsin. Mol. Biol. Int..

[62] Griep M.H., Walczak K.A., Winder E.M., Lueking D.R., Friedrich C.R. (2010). Quantum dot enhancement of bacteriorhodopsin-based electrodes. Biosens. Bioelectron..

[63] Winder E., Lueking D., Friedrich C. Chemical and biological sensing utilizing fused bacteriorhodopsin protein hybrids.

[64] Jovin T.M. (2003). Quantum dots finally come of age. Nat. Biotechnol..

[65] Gerion D., Pinaud F., Williams S.C., Parak W.J., Zanchet D., Weiss S., Alivisatos A.P. (2001). Synthesis and properties of biocompatible water-soluble silica-coated cdse/zns semiconductor quantum dots. J. Phys. Chem. B.

[66] Shibu E.S., Hamada M., Nakanishi S., Wakida S.-I., Biju V. (2014). Photoluminescence of cdse and CdSe/ZnS quantum dots: Modifications for making the invisible visible at ensemble and single-molecule levels. Coordin. Chem. Rev..

[67] Elghanian R., Storhoff J.J., Mucic R.C., Letsinger R.L., Mirkin C.A. (1997). Selective colorimetric detection of polynucleotides based on the distance-dependent optical properties of gold nanoparticles. Science.

[68] Alivisatos P. (2004). The use of nanocrystals in biological detection. Nat. Biotechnol..

[69] Weiss S. (1999). Fluorescence spectroscopy of single biomolecules. Science.

[70] Nagy A., Steinbrück A., Gao J., Doggett N., Hollingsworth J.A., Iyer R. (2012). Comprehensive analysis of the effects of cdse quantum dot size, surface charge, and functionalization on primary human lung cells. ACS Nano..

[71] Wang Y., Hu R., Lin G., Roy I., Yong K.-T. (2013). Functionalized quantum dots for biosensing and bioimaging and concerns on toxicity. ACS Appl. Mater. Interfaces.

[72] Blanco-Canosa J.B., Wu M., Susumu K., Petryayeva E., Jennings T.L., Dawson P.E., Algar W.R., Medintz I.L. (2014). Recent progress in the bioconjugation of quantum dots. Coord. Chem. Rev..

[73] Algar W.R., Tavares A.J., Krull U.J. (2010). Beyond labels: A review of the application of quantum dots as integrated components of assays, bioprobes, and biosensors utilizing optical transduction. Anal. Chim. Acta.

[74] Wang C., Zhao J., Wang Y., Lou N., Ma Q., Su X. (2009). Sensitive Hg (II) ion detection by fluorescent multilayer films fabricated with quantum dots. Sens. Actuators B Chem..

[75] Jin H., Choi S., Velu R., Kim S., Lee H.J. (2012). Preparation of multilayered cdse quantum dot sensitizers by electrostatic layer-by-layer assembly and a series of post-treatments toward efficient quantum dot-sensitized mesoporous TiO_2_ solar cells. Langmuir.

[76] Crawford N.F., Leblanc R.M. (2014). Cdse and cdse(zns) quantum dots in 2d: A langmuir monolayer approach. Coord. Chem. Rev..

[77] Breton B., Sauvageau É, Zhou J., Bonin H., Le Gouill C., Bouvier M. (2010). Multiplexing of multicolor bioluminescence resonance energy transfer. Biophys. J..

[78] De Melo L.S.A., Chaves C.R., Filho P.E.C., Saska S., Nigoghossian K., Gomes A.S., Messaddeq Y., Ribeiro S.J.L., Santos B.S., Fontes A. (2013). Luminescence enhancement of carboxyl-coated cdte quantum dots by silver nanoparticles. Plasmonics.

[79] Chen H., Lin L., Li H., Lin J.-M. (2014). Quantum dots-enhanced chemiluminescence: Mechanism and application. Coord. Chem. Revi..

[80] Stryer L., Haugland R.P. (1967). Energy transfer: A spectroscopic ruler. Biochemistry.

[81] Jares-Erijman E.A., Jovin T.M. (2003). Fret imaging. Nat. Biotechnol..

[82] Noor M.O., Petryayeva E., Tavares A.J., Uddayasankar U., Algar W.R., Krull U.J. (2014). Building from the “ground” up: Developing interfacial chemistry for solid-phase nucleic acid hybridization assays based on quantum dots and fluorescence resonance energy transfer. Coordin. Chem. Rev..

[83] Algar W.R., Kim H., Medintz I.L., Hildebrandt N. (2014). Emerging non-traditional förster resonance energy transfer configurations with semiconductor quantum dots: Investigations and applications. Coord. Chem. Rev..

[84] Wegner K.D., Lanh P.T., Jennings T., Oh E., Jain V., Fairclough S.M., Smith J.M., Giovanelli E., Lequeux N., Pons T. (2013). Influence of luminescence quantum yield, surface coating, and functionalization of quantum dots on the sensitivity of time-resolved fret bioassays. ACS Appl. Mater. Interfaces.

[85] Cassette E., Helle M., Bezdetnaya L., Marchal F., Dubertret B., Pons T. (2013). Design of new quantum dot materials for deep tissue infrared imaging. Adv. Drug Deliv. Rev..

[86] Probst C.E., Zrazhevskiy P., Bagalkot V., Gao X. (2013). Quantum dots as a platform for nanoparticle drug delivery vehicle design. Adv. Drug Deliv. Revi..

[87] Geiβler D., Linden S., Liermann K., Wegner K.D., Charbonnière L.J., Hildebrandt N. (2013). Lanthanides and quantum dots as förster resonance energy transfer agents for diagnostics and cellular imaging. Inorg. Chem..

[88] Mansur A.A.P., Saliba J.B., Mansur H.S. (2013). Surface modified fluorescent quantum dots with neurotransmitter ligands for potential targeting of cell signaling applications. Colloids Surfaces B.

[89] Dennis A.M., Bao G. (2008). Quantum dot-fluorescent protein pairs as novel fluorescence resonance energy transfer probes. Nano Lett..

[90] Frigerio C., Ribeiro D.S.M., Rodrigues S.S.M., Abreu V.L.R.G., Barbosa J.A.C., Prior J.A.V., Marques K.L., Santos J.L.M. (2012). Application of quantum dots as analytical tools in automated chemical analysis: A review. Anal. Chim. Acta.

[91] Wang Y., Chen L. (2011). Quantum dots, lighting up the research and development of nanomedicine. Nanomedicine.

[92] Pichaandi J., van Veggel F.C.J.M. (2014). Near-infrared emitting quantum dots: Recent progress on their synthesis and characterization. Coord. Chem. Rev..

[93] Russ Algar W., Massey M., Krull U.J. (2009). The application of quantum dots, gold nanoparticles and molecular switches to optical nucleic-acid diagnostics. TrAC Trends Anal. Chem..

[94] Kumar M., Zhang D., Broyles D., Deo S.K. (2011). A rapid, sensitive, and selective bioluminescence resonance energy transfer (bret)-based nucleic acid sensing system. Biosens. Bioelectron..

[95] Dacres H., Wang J., Leitch V., Horne I., Anderson A.R., Trowell S.C. (2011). Greatly enhanced detection of a volatile ligand at femtomolar levels using bioluminescence resonance energy transfer (bret). Biosens. Bioelectron..

[96] Hsu C.-Y., Chen C.-W., Yu H.-P., Lin Y.-F., Lai P.-S. (2013). Bioluminescence resonance energy transfer using luciferase-immobilized quantum dots for self-illuminated photodynamic therapy. Biomaterials.

[97] Griep M.H., Winder E.M., Lueking D.R., Friedrich C.R. (2007). An integrated bionanosensing method for airborne toxin detection. Proc. SPIE.

[98] Rui L., Chang Ming L., Haifeng B., Qiaoliang B., Vee S.L. (2007). Stationary current generated from photocycle of a hybrid bacteriorhodopsin/quantum dot bionanosystem. Appl. Phys. Lett..

[99] Rakovich A., Sukhanova A., Bouchonville N., Molinari M., Troyon M., Cohen J.H.M., Rakovich Y., Donegan J.F., Nabiev I. (2009). Energy Transfer Processes in Semiconductor Quantum Dots: Bacteriorhodopsin Hybrid System. Proc. SPIE.

[100] Sternitzke M. (1997). Structural ceramic nanocomposites. J. Eur. Ceramic Soc..

[101] Tjong S.C. (2006). Structural and mechanical properties of polymer nanocomposites. Mat. Sci. Eng. R.

[102] Frogley M.D., Ravich D., Wagner H.D. (2003). Mechanical properties of carbon nanoparticle-reinforced elastomers. Compos. Sci. Technol..

[103] Ounaies Z., Park C., Wise K.E., Siochi E.J., Harrison J.S. (2003). Electrical properties of single wall carbon nanotube reinforced polyimide composites. Compos. Sci. Technol..

[104] Klein D.L., Roth R., Lim A.K.L., Alivisatos A.P., McEuen P.L. (1997). A single-electron transistor made from a cadmium selenide nanocrystal. Nature.

[105] Xia Y.N., Halas N.J. (2005). Shape-controlled synthesis and surface plasmonic properties of metallic nanostructures. Mrs Bull..

[106] Jain P.K., Huang X.H., El-Sayed I.H., El-Sayed M.A. (2008). Noble metals on the nanoscale: Optical and photothermal properties and some applications in imaging, sensing, biology, and medicine. Acc. Chem. Res..

[107] Wang F., Tan W.B., Zhang Y., Fan X.P., Wang M.Q. (2006). Luminescent nanomaterials for biological labelling. Nanotechnology.

[108] Alivisatos A.P. (1996). Semiconductor clusters, nanocrystals, and quantum dots. Science.

[109] Bruchez M., Moronne M., Gin P., Weiss S., Alivisatos A.P. (1998). Semiconductor nanocrystals as fluorescent biological labels. Science.

[110] Chan W.C.W., Nie S.M. (1998). Quantum dot bioconjugates for ultrasensitive nonisotopic detection. Science.

[111] Gao X., Cui Y., Levenson R.M., Chung L.W., Nie S. (2004). *In vivo* cancer targeting and imaging with semiconductor quantum dots. Nat. Biotechnol..

[112] Jain P.K., Lee K.S., El-Sayed I.H., El-Sayed M.A. (2006). Calculated absorption and scattering properties of gold nanoparticles of different size, shape, and composition: Applications in biological imaging and biomedicine. J. Phys. Chem. B.

[113] Lee K.S., El-Sayed M.A. (2006). Gold and silver nanoparticles in sensing and imaging: Sensitivity of plasmon response to size, shape, and metal composition. J. Phys. Chem. B.

[114] De M., Ghosh P.S., Rotello V.M. (2008). Applications of nanoparticles in biology. Adv. Mater..

[115] Huang X.H., Neretina S., el-Sayed M.A. (2009). Gold nanorods: From synthesis and properties to biological and biomedical applications. Adv. Mater..

[116] Lin C.A.J., Lee C.H., Hsieh J.T., Wang H.H., Li J.K., Shen J.L., Chan W.H., Yeh H.I., Chang W.H. (2009). Synthesis of fluorescent metallic nanoclusters toward biomedical application: Recent progress and present challenges. J. Med. Biol. Eng..

[117] Deheer W.A. (1993). The physics of simple metal-clusters—Experimental aspects and simple-models. Rev. Mod. Phys..

[118] Brack M. (1993). The physics of simple metal-clusters—Self-consistent jellium model and semiclassical approaches. Rev. Mod. Phys..

[119] Wilcoxon J.P., Abrams B.L. (2006). Synthesis, structure and properties of metal nanoclusters. Chem. Soc. Rev..

[120] Schmid G., Baumle M., Geerkens M., Helm I., Osemann C., Sawitowski T. (1999). Current and future applications of nanoclusters. Chem. Soc. Rev..

[121] Jin R. (2010). Quantum sized, thiolate-protected gold nanoclusters. Nanoscale.

[122] Shang L., Dong S.J., Nienhaus G.U. (2011). Ultra-small fluorescent metal nanoclusters: Synthesis and biological applications. Nano Today.

[123] Zheng J., Nicovich P.R., Dickson R.M. (2007). Highly fluorescent noble-metal quantum dots. Annu. Rev. Phys. Chem..

[124] Zhang L., Wang E. (2014). Metal nanoclusters: New fluorescent probes for sensors and bioimaging. Nano Today.

[125] Mooradian A. (1969). Photoluminescence of metals. Phys. Rev. Lett..

[126] Schaaff T.G., Knight G., Shafigullin M.N., Borkman R.F., Whetten R.L. (1998). Isolation and selected properties of a 10.4 kda gold: Glutathione cluster compound. J. Phys. Chem. B.

[127] Negishi Y., Takasugi Y., Sato S., Yao H., Kimura K., Tsukuda T. (2004). Magic-numbered Au(n) clusters protected by glutathione monolayers (*n* = 18, 21, 25, 28, 32, 39): Isolation and spectroscopic characterization. J. Am. Chem. Soc..

[128] Wu Z.K., Jin R.C. (2010). On the ligand's role in the fluorescence of gold nanoclusters. Nano Lett..

[129] Link S., Beeby A., FitzGerald S., El-Sayed M.A., Schaaff T.G., Whetten R.L. (2002). Visible to infrared luminescence from a 28-atom gold cluster. J. Phys. Chem. B.

[130] Zheng J., Petty J.T., Dickson R.M. (2003). High quantum yield blue emission from water-soluble au-8 nanodots. J. Am. Chem. Soc..

[131] Zheng J., Zhang C., Dickson R.M. (2004). Highly fluorescent, water-soluble, size-tunable gold quantum dots. Phys. Rev. Lett..

[132] Xie J., Zheng Y., Ying J.Y. (2009). Protein-directed synthesis of highly fluorescent gold nanoclusters. J. Am. Chem. Soc..

[133] Le Guevel X., Hotzer B., Jung G., Hollemeyer K., Trouillet V., Schneider M. (2011). Formation of fluorescent metal (au, ag) nanoclusters capped in bovine serum albumin followed by fluorescence and spectroscopy. J. Phys. Chem. C.

[134] Wei H., Wang Z., Yang L., Tian S., Hou C., Lu Y. (2010). Lysozyme-stabilized gold fluorescent cluster: Synthesis and application as Hg^2+^ sensor. Analyst.

[135] Liu G., Shao Y., Wu F., Xu S., Peng J., Liu L. (2013). DNA-hosted fluorescent gold nanoclusters: Sequence-dependent formation. Nanotechnology.

[136] Lin C.A., Lee C.H., Yeh H.I., Chang W.H., Dössel O., Schlegel W.C. Fluorescent Gold Nanoclusters for Biomedical Applications. International Federation for Medical and Biological Engineering Proceedings.

[137] Lin C.A., Yang T.Y., Lee C.H., Huang S.H., Sperling R.A., Zanella M., Li J.K., Shen J.L., Wang H.H., Yeh H.I. (2009). Synthesis, characterization, and bioconjugation of fluorescent gold nanoclusters toward biological labeling applications. ACS Nano.

[138] Petty J.T., Story S.P., Hsiang J.C., Dickson R.M. (2013). DNA-templated molecular silver fluorophores. J. Phys. Chem. Lett..

[139] Petty J.T., Zheng J., Hud N.V., Dickson R.M. (2004). DNA-templated ag nanocluster formation. J. Am. Chem. Soc..

[140] Richards C.I., Choi S., Hsiang J.C., Antoku Y., Vosch T., Bongiorno A., Tzeng Y.L., Dickson R.M. (2008). Oligonucleotide-stabilized ag nanocluster fluorophores. J. Am. Chem. Soc..

[141] Vosch T., Antoku Y., Hsiang J.C., Richards C.I., Gonzalez J.I., Dickson R.M. (2007). Strongly emissive individual DNA-encapsulated Ag nanoclusters as single-molecule fluorophores. Proc. Natl. Acad. Sci. USA.

[142] Zhang J.G., Xu S.Q., Kumacheva E. (2005). Photogeneration of fluorescent silver nanoclusters in polymer microgels. Adv. Mater..

[143] Wang C., Xu L., Xu X., Cheng H., Sun H., Lin Q., Zhang C. (2014). Near infrared ag/au alloy nanoclusters: Tunable photoluminescence and cellular imaging. J. Colloid Interface Sci..

[144] Goswami N., Giri A., Bootharaju M.S., Xavier P.L., Pradeep T., Pal S.K. (2011). Copper quantum clusters in protein matrix: Potential sensor of Pb^2+^ ion. Anal. Chem..

[145] Tanaka S., Miyazaki J., Tiwari D.K., Jin T., Inouye Y. (2011). Fluorescent platinum nanoclusters: Synthesis, purification, characterization, and application to bioimaging. Angew. Chem..

[146] Tanaka S., Aoki K., Muratsugu A., Ishitobi H., Jin T., Inouye Y. (2013). Synthesis of green-emitting pt-8 nanoclusters for biomedical imaging by pre-equilibrated Pt/pamam (G4-OH) and mild reduction. Opt. Mater. Express.

[147] Le Guevel X. (2014). Recent advances on the synthesis of metal quantum nanoclusters and their application for bioimaging. IEEE J. Sel. Top. Quan..

[148] Zheng J., Dickson R.M. (2002). Individual water-soluble dendrimer-encapsulated silver nanodot fluorescence. J. Am. Chem. Soc..

[149] Peyser L.A., Vinson A.E., Bartko A.P., Dickson R.M. (2001). Photoactivated fluorescence from individual silver nanoclusters. Science.

[150] Balogh L., Tomalia D.A. (1998). Poly(amidoamine) dendrimer-templated nanocomposites. 1. Synthesis of zerovalent copper nanoclusters. J. Am. Chem. Soc..

[151] Zhao M.Q., Sun L., Crooks R.M. (1998). Preparation of Cu nanoclusters within dendrimer templates. J. Am. Chem. Soc..

[152] Rotaru A., Dutta S., Jentzsch E., Gothelf K., Mokhir A. (2010). Selective dsdna-templated formation of copper nanoparticles in solution. Angew. Chem. Int. Edit..

[153] Liu G., Shao Y., Peng J., Dai W., Liu L., Xu S., Wu F., Wu X. (2013). Highly thymine-dependent formation of fluorescent copper nanoparticles templated by ss-DNA. Nanotechnology.

[154] Huang C.C., Yang Z., Lee K.H., Chang H.T. (2007). Synthesis of highly fluorescent gold nanoparticles for sensing mercury(II). Angew. Chem..

[155] Xie J., Zheng Y., Ying J.Y. (2010). Highly selective and ultrasensitive detection of Hg^2+^ based on fluorescence quenching of au nanoclusters by Hg^2+^-au^+^ interactions. Chem. Commun..

[156] Dai H., Shi Y., Wang Y., Sun Y., Hu J., Ni P., Li Z. (2014). Label-free turn-on fluorescent detection of melamine based on the anti-quenching ability of hg^2+^ to gold nanoclusters. Biosens. Bioelectron..

[157] Kawasaki H., Yoshimura K., Hamaguchi K., Arakawa R. (2011). Trypsin-stabilized fluorescent gold nanocluster for sensitive and selective Hg^2+^ detection. Anal. Sci..

[158] Zhang G.M., Li Y.H., Xu J., Zhang C.H., Shuang S.M., Dong C., Choi M.M.F. (2013). Glutathione-protected fluorescent gold nanoclusters for sensitive and selective detection of Cu^2+^. Sens. Actuators B Chem..

[159] Shang L., Dong S.J. (2008). Silver nanocluster-based fluorescent sensors for sensitive detection of Cu(II). J. Mater. Chem..

[160] Chen W.Y., Lan G.Y., Chang H.T. (2011). Use of fluorescent DNA-templated gold/silver nanoclusters for the detection of sulfide ions. Anal. Chem..

[161] Cui M.L., Liu J.M., Wang X.X., Lin L.P., Jiao L., Zheng Z.Y., Zhang L.H., Jiang S.L. (2013). A promising gold nanocluster fluorescent sensor for the highly sensitive and selective detection of S^2−^. Sens. Actuators B Chem..

[162] Zhang H., Liu Q., Wang T., Yun Z., Li G., Liu J., Jiang G. (2013). Facile preparation of glutathione-stabilized gold nanoclusters for selective determination of chromium (III) and chromium (VI) in environmental water samples. Anal. Chim. Acta.

[163] Mu X.Y., Qi L., Dong P., Qiao J., Hou J., Nie Z.X., Ma H.M. (2013). Facile one-pot synthesis of L-proline-stabilized fluorescent gold nanoclusters and its application as sensing probes for serum iron. Biosens. Bioelectron..

[164] Liu Y.L., Ai K.L., Cheng X.L., Huo L.H., Lu L.H. (2010). Gold-nanocluster-based fluorescent sensors for highly sensitive and selective detection of cyanide in water. Adv. Funct. Mater..

[165] Sharma J., Yeh H.C., Yoo H., Werner J.H., Martinez J.S. (2011). Silver nanocluster aptamers: *In situ* generation of intrinsically fluorescent recognition ligands for protein detection. Chem. Commun..

[166] Wen T., Qu F., Li N.B., Luo H.Q. (2012). Polyethyleneimine-capped silver nanoclusters as a fluorescence probe for sensitive detection of hydrogen peroxide and glucose. Anal. Chim. Acta.

[167] Wang M., Mei Q., Zhang K., Zhang Z. (2012). Protein-gold nanoclusters for identification of amino acids by metal ions modulated ratiometric fluorescence. Analyst.

[168] Zhang L., Liang R.P., Xiao S.J., Bai J.M., Zheng L.L., Zhan L., Zhao X.J., Qiu J.D., Huang C.Z. (2014). DNA-templated ag nanoclusters as fluorescent probes for sensing and intracellular imaging of hydroxyl radicals. Talanta.

[169] Wang X., Lin R., Xu Z., Huang H., Li L., Liu F., Li N., Yang X. (2013). *N*-acetylcysteine induced quenching of red fluorescent oligonucleotide-stabilized silver nanoclusters and the application in pharmaceutical detection. Anal. Chim. Acta.

[170] Dou Y., Yang X. (2013). Novel high-sensitive fluorescent detection of deoxyribonuclease I based on DNA-templated gold/silver nanoclusters. Anal. Chim. Acta.

[171] Gao Z., Su R.X., Qi W., Wang L.B., He Z.M. (2014). Copper nanocluster-based fluorescent sensors for sensitive and selective detection of kojic acid in food stuff. Sens. Actuators B Chem..

[172] Qian Y., Zhang Y., Lu L., Cai Y. (2014). A label-free DNA-templated silver nanocluster probe for fluorescence on-off detection of endonuclease activity and inhibition. Biosens. Bioelectron..

[173] Zhou Z.X., Du Y., Dong S.J. (2011). DNA-ag nanoclusters as fluorescence probe for turn-on aptamer sensor of small molecules. Biosens. Bioelectron..

[174] Tao Y., Lin Y., Ren J., Qu X. (2013). A dual fluorometric and colorimetric sensor for dopamine based on bsa-stabilized au nanoclusters. Biosens. Bioelectron..

[175] Van Lith J., Lassesson A., Brown S.A., Schulze M., Partridge J.G., Ayesh A. (2007). A hydrogen sensor based on tunneling between palladium clusters. Appl. Phys. Lett..

[176] Zhang M., Dang Y.Q., Liu T.Y., Li H.W., Wu Y.Q., Li Q., Wang K., Zou B. (2013). Pressure-induced fluorescence enhancement of the bsa-protected gold nanoclusters and the corresponding conformational changes of protein. J. Phys. Chem. C.

[177] Qiang Y., Antony J., Sharma A., Nutting J., Sikes D., Meyer D. (2006). Iron/iron oxide core-shell nanoclusters for biomedical applications. J. Nanopart. Res..

